# Metabolomic Identification of Anticancer Metabolites of Australian Propolis and Proteomic Elucidation of Its Synergistic Mechanisms with Doxorubicin in the MCF7 Cells

**DOI:** 10.3390/ijms22157840

**Published:** 2021-07-22

**Authors:** Muhammad A. Alsherbiny, Deep J. Bhuyan, Ibrahim Radwan, Dennis Chang, Chun-Guang Li

**Affiliations:** 1 NICM Health Research Institute, Western Sydney University, Penrith, NSW 2751, Australia; D.Chang@westernsydney.edu.au; 2 Department of Pharmacognosy, Faculty of Pharmacy, Cairo University, Cairo 11562, Egypt; 3 Faculty of Science and Technology, University of Canberra, Canberra, ACT 2617, Australia; ibrahim.radwan@canberra.edu.au

**Keywords:** doxorubicin, breast cancer, breast adenocarcinoma, MCF7, propolis, synergy, proteomics, metabolomics, apoptosis

## Abstract

The combination of natural products with standard chemotherapeutic agents offers a promising strategy to enhance the efficacy or reduce the side effects of standard chemotherapy. Doxorubicin (DOX), a standard drug for breast cancer, has several disadvantages, including severe side effects and the development of drug resistance. Recently, we reported the potential bioactive markers of Australian propolis extract (AP-1) and their broad spectrum of pharmacological activities. In the present study, we explored the synergistic interactions between AP-1 and DOX in the MCF7 breast adenocarcinoma cells using different synergy quantitation models. Biochemometric and metabolomics-driven analysis was performed to identify the potential anticancer metabolites in AP-1. The molecular mechanisms of synergy were studied by analysing the apoptotic profile via flow cytometry, apoptotic proteome array and measuring the oxidative status of the MCF7 cells treated with the most synergistic combination. Furthermore, label-free quantification proteomics analysis was performed to decipher the underlying synergistic mechanisms. Five prenylated stilbenes were identified as the key metabolites in the most active AP-1 fraction. Strong synergy was observed when AP-1 was combined with DOX in the ratio of 100:0.29 (*w*/*w*) as validated by different synergy quantitation models implemented. AP-1 significantly enhanced the inhibitory effect of DOX against MCF7 cell proliferation in a dose-dependent manner with significant inhibition of the reactive oxygen species (*p* < 0.0001) compared to DOX alone. AP-1 enabled the reversal of DOX-mediated necrosis to programmed cell death, which may be advantageous to decline DOX-related side effects. AP-1 also significantly enhanced the apoptotic effect of DOX after 24 h of treatment with significant upregulation of catalase, HTRA2/Omi, FADD together with DR5 and DR4 TRAIL-mediated apoptosis (*p* < 0.05), contributing to the antiproliferative activity of AP-1. Significant upregulation of pro-apoptotic p27, PON2 and catalase with downregulated anti-apoptotic XIAP, HSP60 and HIF-1α, and increased antioxidant proteins (catalase and PON2) may be associated with the improved apoptosis and oxidative status of the synergistic combination-treated MCF7 cells compared to the mono treatments. Shotgun proteomics identified 21 significantly dysregulated proteins in the synergistic combination-treated cells versus the mono treatments. These proteins were involved in the TP53/ATM-regulated non-homologous end-joining pathway and double-strand breaks repairs, recruiting the overexpressed *BRCA1* and suppressed *RIF1* encoded proteins. The overexpression of *UPF2* was noticed in the synergistic combination treatment, which could assist in overcoming doxorubicin resistance-associated long non-coding RNA and metastasis of the MCF7 cells. In conclusion, we identified the significant synergy and highlighted the key molecular pathways in the interaction between AP-1 and DOX in the MCF7 cells together with the AP-1 anticancer metabolites. Further in vivo and clinical studies are warranted on this synergistic combination.

## 1. Introduction

Doxorubicin (DOX), also known as Adriamycin (a type of anthracycline), is a frontline cytotoxic drug used in numerous chemotherapeutic protocols for various cancer types, including breast cancer [1,2,3,4,5,6]. Despite its broad-spectrum cytotoxic effects [3,7,8,9,10,11,12,13,14], DOX is associated with several severe side effects, including cardiotoxicity, hepatotoxicity, nephrotoxicity and fertility issues. In particular, DOX has been reported to cause lethal cardiomyopathy in cancer patients through free radical-induced oxidative stress and excessive production of reactive oxygen species [15,16]. The type 1 cardiac damage caused by a cumulative dose of doxorubicin is irreversible [17]. Dose-dependent cardiotoxicity of DOX is mediated via interference with DNA replication and transcription, which limits its therapeutic application [15,18]. Additionally, the development of drug resistance of the cytotoxic agents such as DOX poses a considerable challenge in cancer therapy [3,19,20]. Therefore, more efforts are being directed toward a combination therapy or the development of targeted drug delivery formulations to increase DOX therapeutic potential or alleviate adverse effects [11,21,22,23,24,25,26,27,28,29,30].

Combination therapies have been widely adopted to overcome the limitations of the monotherapy regimens and perhaps a compelling approach in cancer treatment that offers benefits via patient-to-patient variability even without drug synergy [31]. The combination strategy may help overcome cancer complexity via targeting multiple pathophysiological components of the disease [32,33]. Combination therapies target different cellular pathways and block cancer evolution escape mechanisms and drug resistance [34]. However, the lack of a gold standard synergy quantitation model warrants considering different synergy metrics to understand the interactions of the individual components in the combination therapy. Different assumptions and limitations in various metrics fueled the persistence of historical rifts among these discording synergy models [35,36]. Various synergy metrics, including Loewe additivity [37], Zero independence potency (ZIP) [38], highest single agent (HSA) [39], and Bliss independence [40], displayed a modest Pearson and Spearman correlation with strong disagreement instances when calculated for O’Neil anticancer combination dataset [36,41,42]. Additionally, substantial disagreements reported when correlating synergy scores originated from different datasets [36]. Therefore, synergistic combinations of interest should be validated against different models before further studies to be considered.

A growing body of evidence demonstrates the advantages of the concurrent administration of herbal medicines with chemotherapy regimens [29,43,44,45,46,47,48] not only as cytotoxic agents but also as an antidote for chemotherapy-induced multi-organ toxicities. Propolis, for example, is a resinous substance accumulated by the bees from different types of plants with a broad spectrum of activities, including antioxidant, anti-inflammatory, antimicrobial and anticancer properties [49,50,51,52]. Australian propolis possesses superior pharmacological activity with a unique chemical fingerprint compared to its Brazilian and Chinese counterparts [49], presumably due to Australia’s megadiverse and unique biodiversity. Previously, prenylated stilbenes isolated from Kangaroo Island propolis [53,54] showed promising antioxidant and cytotoxic properties against 60 human tumour cell lines (NCI-60) with the IC_50_ values 0.68–2.7 µM against the MCF7 cells. Prenylated-flavanones with potential antimicrobial and anticancer activity have also been previously isolated from propolis samples collected worldwide [55,56,57,58,59,60,61,62]. As novel drug development entails ample resources and time, combining pre-existing anticancer drugs with natural product-based adjuvants such as propolis or its metabolites could be a promising and economical approach to enhance the efficacy and/or reduce the side effects of chemotherapy.

The present study was designed to assess the synergistic interactions between Australian propolis (AP-1) and DOX against the MCF7 breast adenocarcinoma cell line using different synergy quantification models. Furthermore, we evaluated the molecular mechanisms involved in the most synergistic combination by analysing the apoptotic profile and oxidative status of the treated MCF7 cells along with the comprehensive biochemometric and metabolomic-driven identification of AP-1 anticancer metabolites. Label-free quantification proteomics analysis was conducted to decipher the complex molecular pathways of the underlying synergistic mechanisms.

## 2. Results and Discussion

### 2.1. Biochemometric and LCMS Metabolomic Identification of Cytotoxic Metabolites of AP-1

We recently evaluated the AP-1 for the potential marker metabolites compared to Chinese and Brazilian propolis samples. In addition, seven common phenolics, including CAPE, artepillin C, galangin, chrysin, pinocembrin, daidzein and naringenin, were quantified in AP-1 using HPLC [49]. In the present study, almost no cell death was observed for normal macrophages (RAW 264.7) upon treatment with AP-1 and its DOX combination up to 200 µg mL^−1^. The IC_50_ value of 177.2 µg mL^−1^ was observed against MCF10A normal breast cell line for AP-1 with a 95% confidence interval of 150.5 to 215.7 µg mL^−1^ (Figure S1). AP-1 showed an MCF7 selectivity index of 2.81 and >2.85 compared with MCF10A and RAW 264.7 cells, respectively. Additionally, a growing body of evidence supports that propolis is generally considered safe [63,64,65,66].

**Figure 1:**
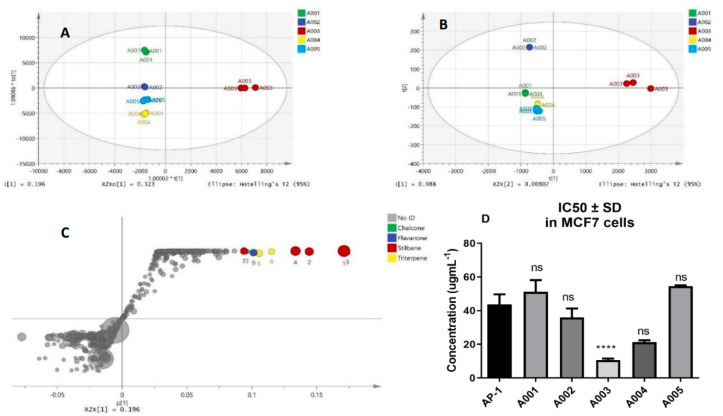
Biochemometric and LCMS metabolomic-driven identification of anticancer metabolites in AP-1 against the MCF7 breast adenocarcinoma cells. (**A**) Score plot of the UPLC-MS (*m*/*z* 50–1200) principal component analyses (PCA) of the significant metabolome of propolis fractions as described by vectors of principal component 1 and 2, (**B**) Score plot of the UPLC-MS (*m*/*z* 50–1200) OPLS-DA selected metabolites of propolis fractions as described by vectors 1 and 2. (**C**) Loading scatter S-plot of the UPLC-MS OPLS-DA analysis of significant AP-1 metabolites, comparing the most active fraction with less active ones, with a legend indicating its chemical class and feature size reflects its abundance in the crude extract. (**D**) Average concentration inhibiting 50% of the MCF7 cells (IC_50_) upon treatment with AP-1 and its fractions for 72 h (*n* = 3, ns = non-significant, **** = statistically significant compared to the propolis extract at *p* < 0.0001 via one-way ANOVA with Dunnet’s correction of multiple comparisons).

AP-1 was subjected to C18 preparative HPLC fractionation into five fractions (A001–A005), and their antiproliferative activity in the MCF7 breast adenocarcinoma cells was evaluated using alamarBlue assay (Figure 1D). The fraction A003 exhibited the most significant cytotoxicity against the MCF7 cells with an IC_50_ value of 10.62 ± 0.88 µg mL^−1^ compared to the other four fractions.

**Figure 2:**
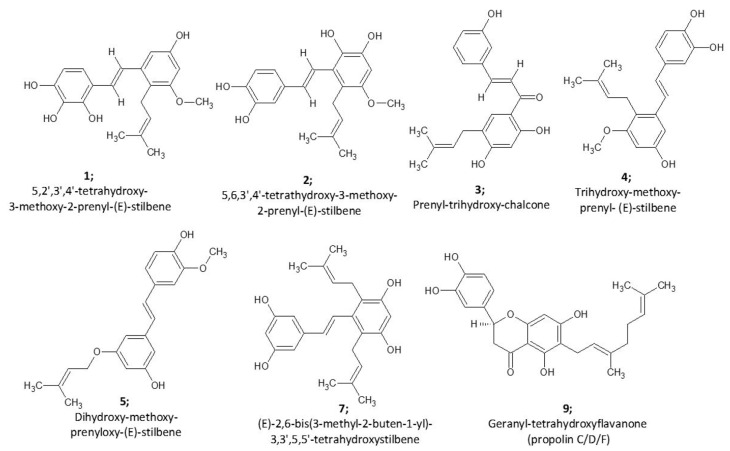
Putative LC-MS identified metabolites in the AP-1 extract with potential anticancer activity against the MCF7 cells.

The LCMS metabolomic profile of A003 was compared with other less active fractions to spot the marker metabolites responsible for the antiproliferative effect. Statistically significant metabolites (ANOVA, *p* ≤ 0.05 and fold change ≥ 2) were subjected to OPLS-Da analysis to identify the discriminating metabolites of the active fraction against others. Nine metabolites were recognized and putatively identified. Notably, good discrimination among propolis fractions was preserved, as shown in the score plots of PCA analyses of the significant metabolome and OPLS-DA-filtered metabolites (Figure 1A,B), despite the features were massively reduced from 1831 to 9. All precursor ions, adducts, fragments, and collision cross-sections (CCS) calculated from ion mobility with the retention time and mass error are listed in Table S1. Five prenylated stilbenes (compounds **1**, **2**, **4**, **5** and **7**) were putatively identified (Figure 1 and Figure 2 and Table S1) with two prenylated flavonoids (flavanone and chalcone) and two undefined triterpenes. The compounds **4**, **5** and **7** were previously isolated from Kangaroo Island propolis, South Australia [53,54] and promising antioxidant and cytotoxic properties were reported against a panel of 60 human tumour cell lines (NCI-60) with the IC_50_ values of 0.68–2.7 µM against the MCF7 cells. Prenylated-flavanones (e.g., compound 9) have also been previously isolated from propolis samples collected from Egypt, Nigeria, Brazil, Indonesia, Japan, Taiwan, Australia and Solomon Islands [55,56,57,58,59,60,61,62] with potential antimicrobial and anticancer activities. Another flavonoid subclass, chalcone, was tentatively identified (compound **3**), sharing common fragments with compound **7**, including *m*/*z* 323.1281, 255.0615 and 254.0567.

Various pharmacological effects of the prenylated chalcones (e.g., compound **3**) have been reported in the literature, including the anticancer activity [67,68,69,70,71]. Compounds **1** and **2** differ from previously isolated and fully characterised prenyl stilbenes such as compounds **4** and **5** in one extra oxygen atom (15.99–16.00 Da) and share their characteristic fragments at *m*/*z* 188.0480, and 144.0580. Therefore, the hydroxylated candidates of isolated prenyl stilbenes (C_20_H_22_O_4_) from AP-1 were prepared, and Competitive Fragmentation Modeling-ID (CFMID 4.0) was utilised for candidate ranking with 10 ppm mass tolerance and both Dice and DotProduct scoring functions were considered [72]. The highest scores were allocated to tetra-hydroxy-methoxy-prenyl stilbenes (5,2′,3′,4′-tetrahydroxy-3-methoxy-2-prenyl-(E)-stilbene and 5,6,3′,4′-tetra-hydroxy-3-methoxy-2-prenyl-(E)-stilbene) upon CFMID-matching with the fragmentation pattern of compound **1** and **2**, respectively (Figure 2).

### 2.2. Synergy Quantification of AP-1 and DOX Combinations against the MCF7 Breast Adenocarcinoma Cells

As there is no gold standard synergy model [42] to quantify the complex synergistic interactions between drugs, we implemented multiple synergy quantification metrics to gain a comprehensive understanding of the potential synergistic interactions between AP-1 and DOX. The Combination Index (CI) model was used to quantify the cytotoxic interactions between AP-1 and DOX in the MCF7 cells after 72 h of treatment. The CI < 1 and CI > 1 indicate synergy and antagonism, respectively, whereas additivity is indicated by CI = 0 [73]. AP-1 and DOX were combined in ratios from 100:2.6 to 100:0.03 *w*/*w*, and CompuSyn-calculated CI values at 50, 75, 90, 95 and 97% inhibitory concentrations were reported in Table 1. Each combination was represented by IDs (e.g., PDOX19), where the last two digits indicate the corresponding combination ratio *w*/*w* of AP-1 and DOX, respectively (Table 1). A strong synergy was observed for the PDOX55 combination (100:0.29 *w*/*w*) in all modelled inhibitory concentrations (Figure S2) where the first dose of 100 µg mL^−1^ AP-1 and 0.29 µg mL^−1^ DOX showed the CI value of 0.11 with 94% cell-growth inhibition. The same data of the AP-1 and DOX combinations were imported to the DrugComb webserver. In addition, checkerboard assay was used to combine DOX and AP-1 in 1:10 and 1:2 serial dilutions, respectively. This enabled synergy quantification in Loewe, ZIP, BLISS, HSA and S synergy score models in addition to the CSS to gain a comprehensive understanding of the synergistic interactions between AP-1 and DOX to inhibit the MCF7 cells.

**Figure 3:**
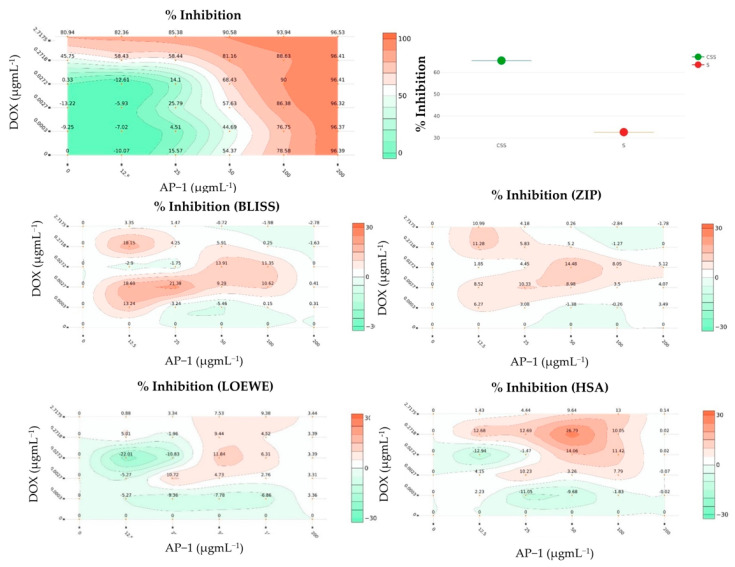
Synergy and sensitivity quantitation of AP-1 in combination with DOX against the MCF7 cells in a checkerboard assay.

Unlike synergy that captures the drug interactions, the combination sensitivity score (CSS) measures the efficacy, and its negligence may lead to biased synergistic combinations [74]. The CSS is a robust metric derived from the relative IC_50_ value and area under the drug combination dose-response curve and was developed for efficacy quantification of drug combinations [75]. Figure 3 and Table 1 summarised the sensitivity and interactions between AP-1 and DOX checkerboard combinations. Notably, potential synergy was observed between AP-1 and DOX in most models with a promising CSS value. Interestingly, both CSS and S scores were able to capture sensitivity and synergy, respectively, for both CI-model data and its combination when reanalysed in DrugComb, unlike other synergy metrics.

**Figure 4:**
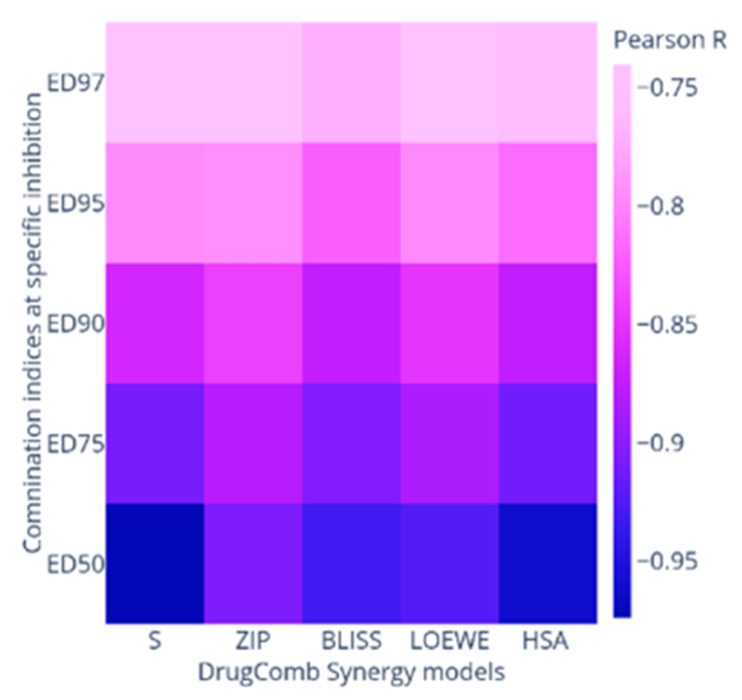
Pearson’s correlation *r* values among different synergy quantitation metrics.

The reprocessing of CI-model data of PDOX combos or their combined responses via DrugComb showed a notable antagonism in all synergy models except for the S synergy score (The increased % inhibition when two drugs are additive at their relative IC_50_). However, strong correlation to the CI-model derived interactions were indicated by Pearson’s correlation *r* values (−0.75:−0.96), where the negative correlation signalled the different scaling where the synergistic potential of CI-model should be < 0 and that for DrugComb synergy scores should be > 0 (Table S2 and Figure 4). Furthermore, the HSA model was able to capture the most synergistic combinations such as PDOX55, PDOX82 and PDOX64. Nevertheless, the HSA score for PDOX91 was not in agreement with that of the CI model. Notably, different synergy metrics, including Loewe, ZIP, HSA, and Bliss, displayed a modest correlation with strong disagreement instances when calculated for O’Neil anticancer combination dataset (22,737 unique combinations) [36,41,42]. Besides, substantial disagreements reported when correlating synergy scores originated from different datasets [36]. So, the selected synergistic dose for subsequent studies was validated against different models (Table 1).

### 2.3. Inhibition of Reactive Oxygen Species (ROS) Production in the MCF7 Cells after Mono and Combined Treatments with AP-1 and DOX

**Figure 5:**
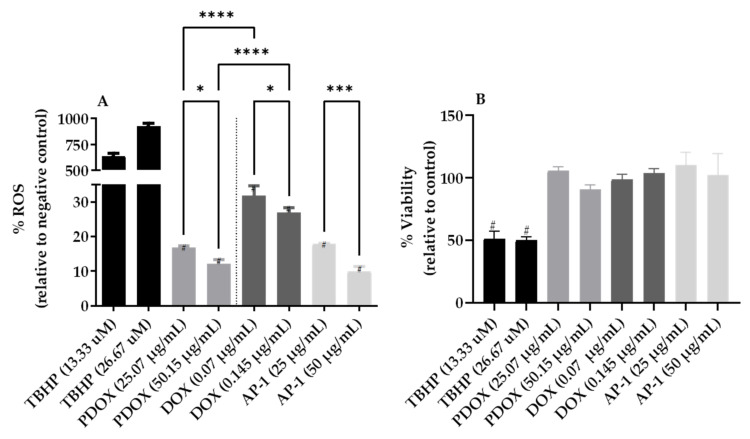
Relative ROS production in MCF7 (**A**) and its percentage viability (**B**) compared to the negative control, upon treatment with Australian propolis (AP-1), doxorubicin (DOX), their synergistic combination (PDOX) and the positive control tert-Butyl hydroperoxide (TBHP). Values expressed as mean ± standard deviation (SD) (*n* = 3), One-way ANOVA was used for multiple comparisons, #; statistically significant relative to negative control (*p* < 0.0001), *; *p* < 0.05, ***; *p* < 0.001, ****; *p* < 0.0001.

Elevated ROS plays a key role in cancer pathogenesis and contributes to tumour metastasis [76,77]. We studied the ROS production in the MCF7 cells treated with AP-1, DOX and their most synergistic combination (as per the tested synergy matrices) in a half and quarter of the selected synergistic dose to avoid any cell death-related ROS depletion. No significant differences in the viability of the MCF7 cells compared to the negative control was indicated for the halved and quartered doses of AP-1, DOX and their combination (Figure 5b).

Furthermore, the ROS production in the MCF7 cells was significantly decreased by AP-1, DOX and their combinations compared to the negative control in a dose-dependent manner. In addition, the combination significantly enhanced the ROS inhibitory effect of DOX (*p* < 0.0001; Figure 5a).

The elevated ROS production and survival dependency were indicated in triple-negative breast cancer cells, which were more sensitive to antioxidant treatments compared to positive estrogen receptor (ER+) cell lines [78]. However, improved MCF7 sensitivity to DOX in combination with an antioxidant such as vitamin C was reported in both noncytotoxic and moderately cytotoxic vitamin C doses [79]. The same enhancement was reported for DOX with vitamin C against the triple-negative MDA-MB-231 cell line which contradicts the finding of Sarmiento-Salinas, et al. [78]. While the ROS decline after AP-1 treatment can be attributed to its antioxidant properties, the DOX-mediated ROS decline observed in our study is inconsistent with the well-documented DOX-induced ROS in cancers and normal cells [80,81]. This may be ascribed to the low DOX doses (0.145 and 0.07 µg mL^−1^) implemented in our study compared to the higher DOX doses reported in the literature or shorter exposure time (4 h). For instance, 40 µg mL^−1^ of DOX after 8 h of treatment was shown to increase the ROS production in the MCF7 cells [82]. However, a 0.3–5.0 µM plasma concentration of DOX is commonly used clinically with a general initial plasma concentration of 1–2 µM DOX can decline quickly to 25–250 nM level within 1 h [81]. Therefore, studies that utilise higher doses (>1–2 µM) of DOX may not accurately reflect the clinical implementation of DOX [81]. In addition, the observed ROS decline in the DOX-treated MCF7 cells in our study may indicate the predominance of DNA synthesis inhibition mechanism rather than a free radical generation with the studied doses. Another study observed no ROS-mediated DCF fluorescence in the PA-1 human ovarian teratocarcinoma cells treated with 0.5 µM DOX compared to the negative control where DOX did not show H_2_O_2_ generation to any extent in the PA-1 cells unlike in the Bovine Aorta Endothelial Cells (BAECs) [83]. The findings of that study were also inconsistent with the literature, however, the authors did not perform fluorescence quantification to measure any ROS decline in the PA-1 cells [83] unlike our study. We used the same dye and same exposure time of 4 h but with lower DOX doses.

Notably, antioxidants have differential effects on DOX-mediated apoptosis and caspase 3 activity in normal and tumour cells where apoptosis and caspase 3 activity declined in BAEC and ARCM normal cells and increased in the MCF-7 and the PA-1 cells [83], which support the use of AP-1 in combination with DOX. Further validations are necessary to support any biphasic dose- and/or time-dependent ROS production profile of DOX. The complex, context-dependent and paradoxical roles of ROS in cancer are well-reported in the literature with ROS surge linked to both the tumour proliferative processes and a potential avenue to selectively target cancer cells [84]. For example, piperlongumine [85] and blueberry extracts [86] selectively induced ROS in cancer cells, but not in the normal MCF10A cells. Section 2.5 will explore the dysregulated apoptotic antioxidant proteins in the MCF7 cells upon the combination and mono treatments and their paradoxical effects.

### 2.4. Flow Cytometric Analysis of Apoptosis in the MCF7 Cells Using Annexin V-CF Blue and 7AAD

Synergistic interactions between anticancer drugs are desirable traits to enhance efficacy, reduce dosage and mitigate the subsequent adverse effects. This strategy is also promising in overcoming the escape mechanisms and drug resistance of cancer [32,33,34]. In this study, we evaluated the apoptotic profiles of the MCF7 cells using flow cytometry after treatment with AP-1 (100 µg mL^−1^), DOX (0.29 µg mL^−1^) and their most synergistic combination PDOX55 (At half-dose; 50 µg mL^−1^ AP-1 and 0.15 µg mL^−1^ DOX). Simultaneous evaluation of the live, early to late apoptotic and necrotic cell populations was carried out to observe whether the most synergistic combination had any effect on apoptotic pathways of the MCF7 cells compared to mono treatments. The half-dose of the most synergistic combination was implemented to statistically evaluate the effects of the combined treatment as higher doses might lead to elevated apoptosis via additive effect, however with side effects. Annexin V is commonly used to detect apoptosis by binding to the phosphatidylserine (PS) phospholipids on the cell surface. PS is translocated to the outer surface of cells during apoptosis [87]. Conversely, 7-AAD is a fluorescent dye that intercalates in double-stranded DNA with a high affinity for guanine–cytosine residues and is used as a DNA fluorescent marker in flow cytometry and fluorescence microscopy [88,89].

**Figure 6:**
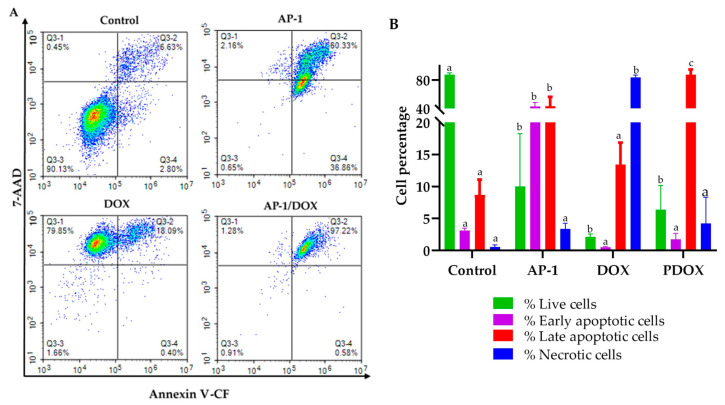
(**A**) Flow cytometric assessment of apoptotic profiles of the MCF7 breast cancer cell line and the images are representative of three separate experiments, (**B**) Cell percentage analysis in different treatment groups in quadruplicates. The AP-1 (100 µg mL^−1^), DOX (0.29 µg mL^−1^) and their most synergistic combination (at half-dose; 50 µg mL^−1^ AP-1 + 0.15 µg mL^−1^ DOX) with the vehicle control were implemented using antibodies against Annexin-V CF-Blue and the reporter 7AAD after 24 h of treatment. Superscript letters indicate statistical significance derived from two-way ANOVA and Tukey’s multiple comparisons within the same cell group (bar colour) where different letters are statistically significant with *p* < 0.0001 (*n* = 4). Raw data are available in Supplementary Table S3.

In apoptotic analysis, Annexin V and 7-AAD are combined to distinguish necrotic cells from early and late apoptotic cells. The PerCP and Pacific blue channels were utilised for Annexin V and 7-AAD in this study as emission spectra of these dyes do not overlap, so no compensation is necessary (Figure S3). After 24 h, significant differences among the live and late apoptotic cell populations were observed in the mono and combined treatments compared to the vehicle control (*p* < 0.0001; *n* = 4) (Figure 6, Figure S4, and Table S3). The AP-1 treatment led to a significant increase in the early and late apoptotic cells (43.02 ± 5.46 and 43.53 ± 12.89, respectively; *p* < 0.0001), whereas the DOX treatment exhibited a significant increase of the necrotic cells (83.85 ± 3.15%; *p* < 0.0001) compared to the vehicle control (Figure 6B, Table S3). Interestingly, the synergistic combination at its half-dose significantly increased the percentage of late apoptotic cells to 87.59 ± 7.44% compared to the vehicle control and the mono treatments. In addition, a significant reduction of the necrotic cells (4.25 ± 4.04%; *p* < 0.0001) was observed for the synergistic combination compared to DOX alone. The number of necrotic cells in the combined treatment was statistically similar to that of the vehicle control, which might indicate the ability of the synergistic combination to shift DOX-mediated necrosis to apoptosis. The observed necrotic to apoptotic shift in the synergistic combination may be attributed to the antioxidant profile of AP-1 [49]. The antioxidant-related apoptotic proteins in the MCF7 cells will be discussed in the Apoptotic Proteome Array analyses (Section 2.5). The necrotic to apoptotic shift has been reported earlier in the literature by other antioxidants [90,91] and was observed in the MCF7 cells treated with DOX and all-trans retinoid acid [92]. Sugimoto, et al. studied the DOX-induced necrosis of Jurkat cells and its acceleration and conversion by antioxidants to apoptosis [93]. Collectively, AP-1 enhanced the anticancer activity of DOX by promoting apoptosis and reducing necrosis which might be advantageous to reduce the DOX-mediated side effects.

### 2.5. Apoptotic Proteome Profiler Array Analysis

#### 2.5.1. Effects of AP-1 and DOX on Apoptotic Proteins of the MCF7 Cells

**Figure 7:**
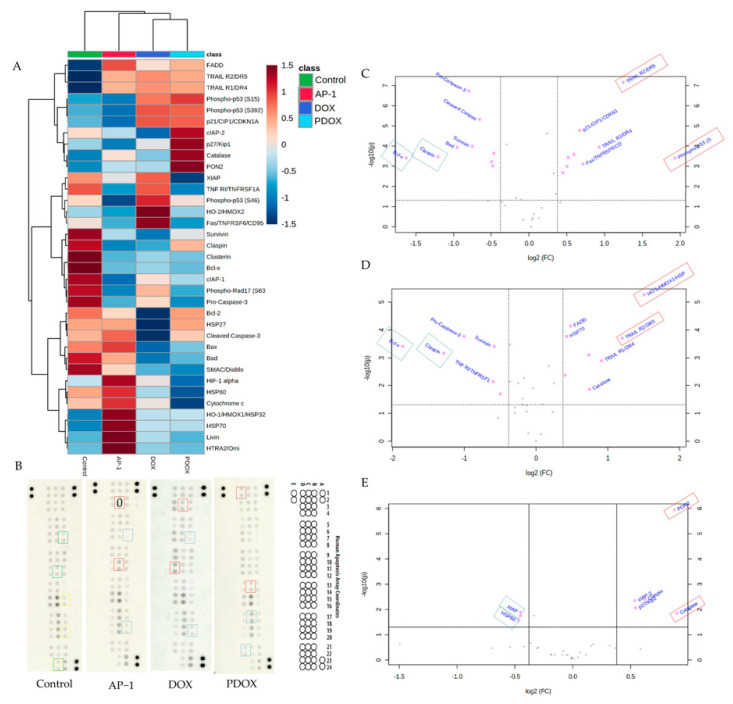
Differently expressed apoptotic proteins in the MCF7 cell lysates after treatment with AP-1, DOX, and the synergistic combination (PDOX). (**A**) Mean apoptotic proteins expression heatmap of the proteome arrays processed data after treatment of MCF7 cells with the vehicle (0.5% DMSO), 0.29 µg mL^−1^ DOX, 100 µg mL^−1^ AP-1 and their synergistic combination with hierarchical clustering of the groups using the Euclidean distance measure and Ward clustering algorithm. (**B**) The MCF7 lysates were analysed by Proteome Profiler^TM^ human apoptotic arrays after 24 h of treatment. The significant features are marked with blue and red rectangles (other than the vehicle control), indicating the downregulation and upregulation of proteins, respectively. The yellow and green rectangles on the control array indicate the significant proteins identified by coefficient and VIP scores of the PLS-DA model, respectively. Protein coordinates are listed in Table S4. (**C**) The significantly dysregulated apoptotic proteins after DOX treatment as selected by volcano plot compared to the control with the fold change (FC) threshold (x) 1.3 and *t*-test threshold (y) 0.05. (**D**) The significantly dysregulated apoptotic proteins after AP-1 treatment as selected by volcano plot compared to the vehicle control with the FC threshold (x) 1.3 and *t*-test threshold (y) 0.05. (**E**) The significantly dysregulated apoptotic proteins after treatment with the synergetic combination (100 µg mL^− 1^ AP-1 and 0.29 µg mL^−1^ DOX) as selected by volcano plot compared to the vehicle control with the FC threshold (x) 1.3 and *t*-test threshold (y) 0.05. The fold changes and *p* values are log-transformed, and the further the FC values are from the (0,0), the more significant the feature is.

The proteome profiler^TM^ human apoptosis array kit was used to study the effect of AP-1 and DOX treatments on 35 apoptotic proteins of the MCF7 breast adenocarcinoma cells. The differential expression of the apoptotic proteins after mono and combined treatments are shown in Figure 7A,B. Figure 7A shows the mono treatments and combination in distant clusters from the control using hierarchical clustering on the top of the heatmap with the help of Euclidean distance measure and Ward clustering algorithm. Furthermore, both unsupervised principal component analysis (PCA) and supervised partial least square discriminant analysis (PLS-Da) indicated the distinct clustering of different treatments and the synergistic combination away from the control based on the profile of apoptotic proteins (Figure S5A,B). The corresponding proteins for the array coordinates are listed in Table S4. Livin, HO-1/HMOX1/HSP32, and Bcl-X, respectively, are identified as the most discriminatory apoptotic proteins among the treatments by variable importance projection (VIP) scores in the constructed PLS-Da model (Figure S5C, red rectangles on the control array Figure 7B). However, PLS-Da coefficient scores outlined the significance of claspin, livin and catalase, respectively, in the classification model (Figure S5D, blue rectangles on the control array Figure 7B). Both scores identified livin as a characteristic protein among different MCF7 cell lysates in this study.

The differentially expressed proteins were selected if their *p*-value was ≤0.05 with an absolute fold change of 1.3 in the pairwise comparisons (Figure 7C,D and Table S5). The top two downregulated or upregulated proteins in the AP-1 and DOX treatment groups (based on fold change compared to the vehicle control) were marked on the arrays by blue and red rectangles, respectively (Figure 7B). These dysregulated proteins are also indicated in the volcano plot Figure 7C,D, where upregulated and downregulated proteins are located on the right and left parts away from the central volcano plot axis (0,0), respectively. Thus, more significant proteins are positioned away from the centre and the bottom part of the volcano plot.

Bcl-x, claspin, pro-caspase-3, survivin, and cIAP-2 were significantly downregulated in the MCF7 cells after mono treatment with AP-1 and DOX. Bcl-x and claspin were the top-two downregulated proteins (Figure 7A–D and Table S5). Bcl-x is a dominant apoptosis regulator in mammalian cells with the long-form (Bcl-xL) responsible for anti-apoptotic effects, and the short isoforms (Bcl-xS and Bcl-xb) promote apoptosis. Bcl-x belongs to the Bcl-2 (B-cell lymphoma 2) family, which can exert either anti-apoptotic or pro-apoptotic effects and is recognised among pro-survival Bcl-2 subfamily members alongside Bcl-w and Mcl-1 [94]. The Bcl-2 subfamily of proteins can promote cell survival by inhibiting the activation of the caspases [94]. Interestingly, the downregulated Bcl-x expression was previously reported as a possible indicator of chemoresistance in myeloma [95] and an inhibitor of Fas-mediated apoptosis in the MCF7 cells [96]. The Bcl-2 level was significantly downregulated in the MCF7 cells after treatment with DOX. Parallel observations were made previously where DOX and etoposide conferred antiproliferative effect via the downregulation of Bcl-2 expression in the MCF7 cells [97]. Induction of autophagy in the MCF7 cells was also observed earlier by Bcl-2 silencing via siRNA [98]. Additionally, Bad (Bcl-2 associated agonist of cell death) was downregulated by DOX. Bad is an anti-apoptotic or pro-apoptotic member of the Bcl-2 family depending on its serine 75, 99 and 118 phosphorylation state [99].

Claspin is an essential component for the ATR-Chk1-dependant activation of the DNA replication in human cells [100,101]. Recently, claspin overexpression was reported to protect the HCT116 cells from replication stress in a checkpoint independent manner [102]. Both AP-1 and DOX significantly suppressed the expressions of claspin and survivin. Claspin is usually overexpressed in almost all malignancies with proliferating and anti-apoptotic activity [103]. Taken together, the downregulated claspin and survivin contributed to the DOX and AP-1 -mediated apoptosis in the MCF7 cells. Previously, DOX was reported to induce cell death in the MCF7 and the MDMB231 breast cancer cells regardless of the expression level of survivin [104].

The expressions of anti-apoptotic proteins such as Bcl-2 and survivin were also correlated to the *HER-2* expression in the MCF7 cells [105]. The *HER-2* oncogene is considered a relevant biomarker and an essential target for approximately 30% of breast cancer patients [106].

The inhibitors of apoptosis (IAPs) are overexpressed in breast cancer cell lines (MCF7 and MDA-MB-231) [107,108,109] and breast cancer patients [110,111]. AP-1 and DOX significantly downregulated the expressions of two IAPs, namely, the cIAP-1 and cIAP-2 (Figure 7A).

The caspase-3 deficiency in the MCF7 was reported to contribute to its chemotherapeutic resistance, where its expression in the MCF7 cells increased the DOX efficacy [112]. Discrepant detection of caspase 3 in the MCF7 cells [113,114,115,116,117] concerns amid *CASP-3* partial deletion and the lack of caspase-3 expression reported in the MCF7 cells [118,119]. Such contradictory findings may be partially explained by using inappropriate antibodies that cross-react with other caspase-3-unrelated proteins or cross-reactivity on fluorogenic substrates, especially with caspase-7 and cathepsin B [119,120]. In the current study, pro-caspase-3 expression was significantly downregulated in the MCF7 cell upon AP-1 and DOX treatments. However, the cleaved caspase-3 (the active form of caspase-3 responsible for apoptotic signal) was found to be downregulated in the DOX treatment group compared to the vehicle control (Figure 7A,B), which might be inconsistent with the downregulation of Bcl-2. The activated caspases are responsible for the cleavage (downregulation) of Bcl-2 [121].

Clusterin is an apoptosis inhibitor that exerts its effect via its interaction with the activated Bax [122] and is considered a key component for chemoresistance [123], where its inhibition sensitised the MCF7 and MDA-MB-231 cells to chemotherapy [124]. Clusterin was significantly suppressed by Dox treatment of the MCF7 cell in the current study.

The loss of p53 pro-apoptotic functions was reported to be associated with the resistance of MCF7 cells to TNF-induced cytotoxicity [125,126,127]. However, the apoptotic activity of AP-1 may not p53-related as the Phospho-p53 (S392) was significantly suppressed (*p* < 0.05), and other Phospho-p53 including S15 and S46 were also downregulated, although not statistically significant compared to the vehicle control (*p* > 0.05). Previously, upregulation of p-53 was reported in the MCF7 cells after treatment with Chinese [128] and Turkish propolis [129]. Our recent study showed the discriminatory metabolites of AP-1 vs. Chinese and Brazilian samples, indicating a greater antiproliferative effect of AP-1 against the MCF7 and MDA-M-B231 cells [49]. The discrepancies in the underlying cytotoxic mechanisms may be correlated to different key metabolites in the propolis samples, accounting for their differential effects on p53 expression. In contrast, the apoptotic effect of DOX in the MCF7 cells was mediated by increased phosphorylated p-53 proteins, including phospho-p53 (S15) and phospho-p53 (S15). These findings are in line with the previous reports indicating the involvement of the p-53 signal transduction pathway in DOX-induced apoptosis [130,131,132,133].

The other apoptotic proteins- Fas and p21 were significantly upregulated in the DOX treatment group, in agreement with previous studies [133,134,135,136]. Furthermore, the MCF7 cell death was mediated by upregulation of TRAIL (TNF-related apoptosis-inducing ligand) R1/DR4 and TRAIL R2/DR5 expression by both AP-1 and DOX. In the current study, the (TRAIL)-mediated apoptosis is confirmed for DOX and AP-1, although the TNF RI/TNFRSF1A (Tumour necrosis factor receptor superfamily member 1A) was downregulated in the latter. The TRAIL-mediated apoptosis was also reported previously for DOX [137,138,139]. The TNFRSF1A is a member of the TNF receptor superfamily and one of the central receptors for TNF-α, and its gene knockout was demonstrated to induce apoptosis in triple-negative breast cancer cells [140].

Heme oxygenases, including HMOX-1 and HMOX-2, were upregulated after DOX treatment, while HMOX1 only was upregulated in the AP-1 treatment group. The ROS generation from the redox cycling of DOX was found to be responsible for its cytotoxicity. However, the Nrf2 signalling pathway activation was reported as a chemoprotective mechanism against DOX and liable for its inclined resistance [141,142]. The DOX and AP-1 treatments also upregulated livin in the MCF7 cells, an IAP, and its surge may account for drug resistance and tumour progression [143]. HIF-1α and HSP-70 have been overexpressed as a defence mechanism against propolis-induced cytotoxicity. These proteins have been reported to mediate metastasis and inhibit cancer cell apoptosis [144,145,146,147].

Heat shock proteins, including four HSP70s (Hsp70-1, Hsc70, Grp75, and Grp78), were the most abundant in MCF7 and associated with estrogen receptor alpha (ERα) followed by HSP90 and HSP110 and thereby effectively able to regulate the ER-mediated cell proliferation [147,148]. Catalase is a critical antioxidant enzyme that metabolises H_2_O_2_ and reactive nitrogen species, and in tumours, its expression and localization are significantly dysregulated [149]. It was reported to be overexpressed in resistant cancer cells, and its downregulation can lead to enhanced cytotoxicity of these cells [150]. However, the upregulated catalase expression suppressed the chemically induced colon cancer in mice model [151] and reduced breast cancer invasiveness and metastasis in the transgenic mice model expressing mitochondrial catalase [152]. Several reports also portrayed the decreased catalase expression in cancer and other diseases [153,154,155,156,157,158,159,160,161,162,163,164,165]. In an earlier study, the proliferation and migration of the MCF7 cells were reduced via catalase overexpression with increased sensitivity to chemotherapy, including etoposide and paclitaxel [166].

The apoptotic effect of AP-1 was also regulated via inclined HTRA2/Omi and FADD (FC = 1.66, and 1.4, respectively) together with the TRAIL-mediated apoptosis. FADD is involved in the Fas signal transduction and reported for its Fas-induced apoptosis in the MCF7 cells [167], and its JNK-mediated phosphorylation was demonstrated to play a critical role in the regulation of cell cycle, cellular growth and metastasis and was not dependent on the ER status of breast cancer [168]. Additionally, the activation of caspase-dependent mitochondrial pathways was reported earlier via the overexpression of the pro-apoptotic HTRA2/Omi [169,170,171].

Altogether, the significant overexpression of catalase, HTRA2/Omi, FADD with TRAIL-mediated apoptosis with 2.3, and 1.89 FC for DR5 and DR4, respectively, provide an insight into the cytotoxic mechanisms of AP-1 against the MCF7 cells.

#### 2.5.2. Effects of the Most Synergistic AP-1 and DOX Combination on Apoptotic Proteins of the MCF7 Cells

The most synergistic combination (100 µg mL^−1^ AP-1 and 0.29 µg mL^−1^ DOX) significantly downregulated the expression of XIAP, HSP60, Cytochrome c, and HIF-1α (0.71–0.77 FC), and upregulated cIAP-2, p27/Kip1, claspin, PON2, and catalase (1.5–2.04 FC) as shown in Figure 7 and Table S5. Overall, the synergistic combination mediated the apoptosis of the MCF7 cells by downregulating anti-apoptotic proteins in addition to the paradox effect of antioxidant paraoxonase 2 (PON2) and catalase.

The overexpression of XIAP was previously found to be associated with breast cancer survival and chemoresistance [97,172]. Its downregulation might lead to enhanced MCF7 sensitivity. The HSP-60 is a mitochondrial protein with pro-survival and anti-apoptotic effects upon binding with survivin in the mitochondria where the survivin is stabilised, and cancer cell survival is achieved [173,174]. The downregulation of HSP60 observed in our study after treatment with the AP-1 and DOX combination might facilitate caspase-dependent apoptosis via destabilising survivin, inducing mitochondrial dysfunction and enhancing p53 function [173,174].

The paradoxical effects of PON2 have been reported earlier, as its overexpression was suggested to prevent the formation of ovarian tumours in the mouse xenograft model of ovarian cancer [175]. The cytotoxic effect against ovarian cancer was demonstrated to be mediated through the increased PON2 expression, which downregulated the expression of insulin-like growth factor-1 (IGF-1) by its antioxidant-related decline in cholesterol production. As a result, cholesterol was not available as a substrate for estrogen production in ovarian cancer cells. The PON2-dependent downregulated expression of IGF-1 and suppressed estrogen production in the ovaries may also be beneficial for breast cancer treatment [176,177,178,179]. In addition, similar to the inhibition of ovarian tumours, the PON2-mediated inhibition of breast cancer can be speculated as both cancers share common etiology with *BRCA1* and *BRCA2* mutations and elevated estrogen levels [180,181,182]. The second significantly upregulated protein in the MCF7 cells after the treatment with the synergistic combination was catalase, and its antiapoptotic effect against the MCF7 cells was discussed in Section 2.5.1.

The increased expression of cytochrome c has been correlated with the apoptotic activity of cells and cells undergoing apoptosis in vivo, where cytochrome c was found to be released to their cytosol [183]. Notably, Bcl-xL was reported to block cytochrome c release from the mitochondria into the cytosol preventing its apoptotic effect [184] and the tyrosine residue (Tyr48) phosphorylation by cytochrome c-phosphorylating kinase impairs Apaf-1-mediated caspase activation, where cytochrome c acts as an anti-apoptotic switch [185]. However, as observed in the flow cytometry data, the synergistic combination at its half-dose resulted in 87.59 ± 7.44% of the MCF7 cells in the late apoptotic stage after 24 h. This might indicate that the release of cytochrome c and subsequent activation of caspase cascade leading to early apoptosis took place earlier than 24 h, and hence, cytochrome c was not found to upregulated in the Apoptosis proteome profiler array analysis.

Cyclin-dependent kinase inhibitor 1B (p27/Kip1) is a tumour suppressor and cell cycle inhibitor protein that regulates the cell cycle progression at the G1 phase via hindered activation of cyclin E-CDK2 or cyclin D-CDK4 complexes [186,187]. Furthermore, declined p27 in breast cancer cells was correlated with oncogenic kinase Src activation, which accelerates p27 proteolysis [188]. Therefore, the significant p27/Kip1 overexpression by the synergistic combination (FC = 1.5, *p*-value = 1.031 × 10^−3^ and FDR = 9.018 × 10^−3^) in the MCF7 cells compared to the mono treatments may contribute to the observed synergistic effects.

These findings encourage the implementation of the synergistic combination of AP-1 and DOX in either estrogen or progesterone receptor-negative breast cancers [188] and *BRCA1/2* mutated breast cancers [189] with low levels of p27 expression.

Altogether, the enhanced apoptotic activity found in the flow cytometry analyses of the synergistic combination compared to the mono treatments against the MCF7 cells may be associated with upregulated expressions of pro-apoptotic p27, PON2 and catalase and downregulation of anti-apoptotic XIAP, HSP60 and HIF-1α. Furthermore, the enhanced antioxidant proteins in the MCF7 cells after treatment with the synergistic combination may be associated with the shift of the DOX-induced necrosis into programmed cell death observed in the flow cytometry analysis (Figure 6).

### 2.6. Bottom-Up Label-Free Quantification Proteomic Study of the MCF7 Cells after Treatment with AP-1, DOX and Their Synergistic Combination

**Figure 8:**
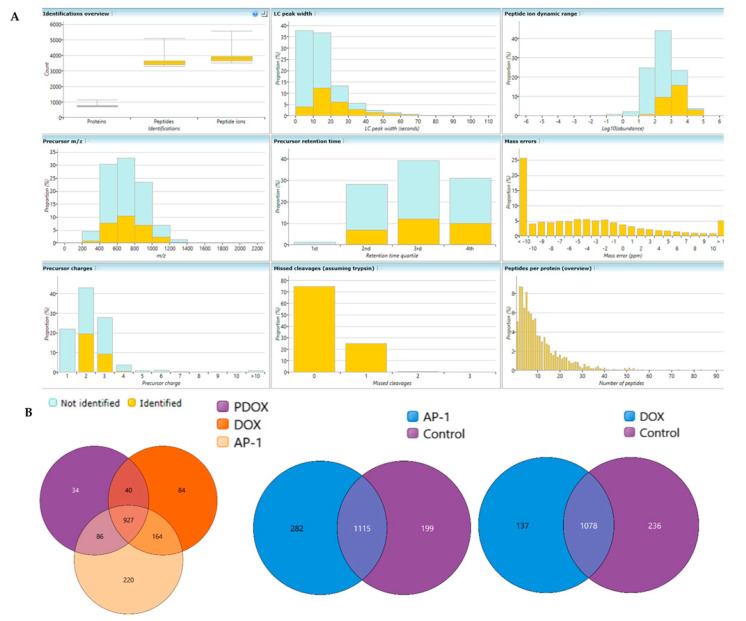
(**A**) Overview of quality control metrics of LC-MS/MS shotgun proteomic study of the MCF7 cells after treatment with AP-1, DOX and their synergistic combination (PDOX). (**B**) Venn diagrams of the overlapped identified proteins in the differently treated groups.

A discovery study on the MCF7 cells treated with AP-1, DOX and their synergistic combination was performed using label-free LC-MS/MS proteomics. The differently expressed proteins in the MCF7 cells belonging to the three treatment groups were analysed in pairwise comparison to the vehicle control or the mono treatments in case of the synergistic combination. The peptide counts, unique peptide counts, *m*/*z* of the identified 1687 proteins, the confidence scores and the statistics, and the fold change (FC) calculations are listed in different worksheets of Supplementary File 2, together with quality control metrics and overlapped proteins among experimental groups shown in Figure 8. Peptides with an absolute mass error of 20 ppm were omitted from the study based on the mass error distribution of the identified peptides (Figure 8A). The differentially expressed proteins in the synergistic combination-treated cells were selected based on the ANOVA test, *p* and q-values of ≤ 0.01 (q-values are the adjusted *p*-values based on the optimised false discovery rate (FDR) approach) with an absolute FC ≥ 1.7. Six upregulated, and fifteen downregulated proteins were identified in the synergistic combination group compared to the averaged protein expression in mono treatment groups (Table 2). These 21 dysregulated proteins display the proteome-level variance acquired by combining AP-1 and DOX. Therefore, it may reflect the possible underlying synergistic mechanisms of action against the MCF7 breast adenocarcinoma cells.

**Figure 9:**
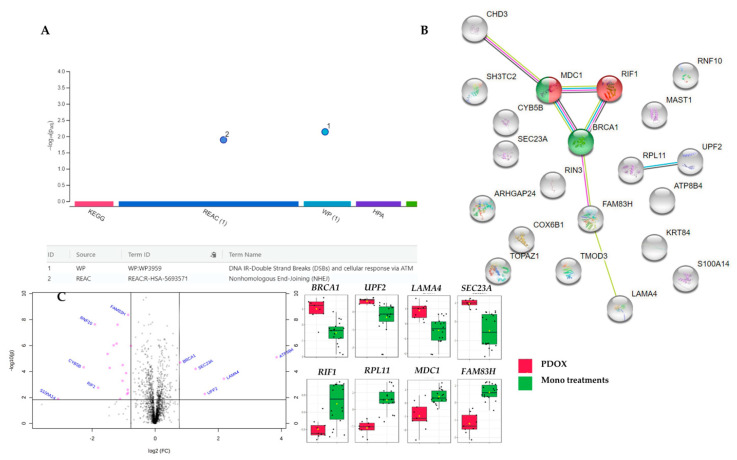
(**A**) Enriched pathways using g:Profiler, (**B**) STRING network of the differentially expressed proteins in the synergistic combination-treated MCF7 cells and (**C**) Volcano plot of 0.015 *p*-value and absolute 1.7 FC threshold among identified proteins in the synergistic combination-treated cells with selected proteins expression summary (PDOX = synergistic combination of AP-1 and DOX). BRCA1-A complex and BRCT domain associated proteins in red and green, respectively, in STRING network. WP; Wikipathways, REAC; Reactome.

When the set of dysregulated proteins of the synergistic combination-treated MCF7 cells was considered, g:Profiler identified a subset of proteins-encoding genes such as *MDC1*, *RIF1,* and *BRCA1* involved in both Nonhomologous End-Joining (NHEJ), and DNA ionising radiation (IR)-double-strand breaks (DSBs) and cellular response via ataxia-telangiectasia mutated (ATM) (Figure 9A and Table 3). The NHEJ pathway was also overrepresented in the Reactome analysis and confirmed with DSB-repair via STING analysis showing a network of interactions involving additional genes such as *CHD3*, *FAM83H* and *LAMA4* (Figure 9B). Genes such as *MDC1*, *BRCA1*, *CHD3*, and *COX6B1* were involved in the transcriptional regulation by *TP53* as significantly identified by Reactome (Table 3 and Table S6) and IMPaLA (Table S7). Other pathways were spotted by Reactome with the *p*-value < 0.05, but high FDR values (0.1–0.16) such as cell cycle checkpoints (*MDC1*, *MAST1* and *BRCA1*), G_2_/M DNA damage checkpoint (*MDC1* and *BRCA1*) and nonsense-mediated decay (*UPF2* and *RPL11*; both were also linked in STRING network).

The breast cancer type 1 susceptibility protein encoded by *BRCA1*, a tumour suppressor gene, was significantly upregulated with 1.77 FC in the synergistic combination group compared to averaged mono treatments (Figure 9C). *BRCA1* mutations are responsible for 40% and 80% of inherited ovarian and breast cancers, respectively. Downregulated or undetectable levels of *BRCA1* expression were reported in most high-grade ductal breast cancers [190] and MCF7 cells [191]. The downregulation of *BRCA1* has been shown to contribute to sporadic and inherited breast cancer progression [192] due to the declined repairs of DNA damage and double-strand breaks with increased gross chromosomal rearrangement and mutations leading to breast cancer progression [193]. *BRCA1* is associated with *RIF1* and *MDC1* in NHEJ and with *MDC1* only in *TP53* transcriptional regulation of DNA repair genes (Figure S6) and DNA double-strand breaks-repairs pathways as identified in different pathway enrichment platforms (Table 3). NHEJ pathway is initiated as a response to DNA-damaging agents causing DNA double-strand breaks with subsequent ATM activation and *MDC1* recruitment for the formation of nuclear foci with the recruitment of DNA damage checkpoints and repairs [194,195,196,197,198]. Ultimately, *BRCA1* and *TP53BP1* are recruited which is crucial for ATM-mediated CHECK2 activation and DNA repair [199,200]. *RIF1* and *PAX1IP* were reported to prevent resection of DNA double-strand break needed for homologous recombination repair via replacing *BRCA1:BARD1* and associated proteins in the DNA double-strand breaks [201,202]. Altogether, AP-1 significantly synergised doxorubicin against the metastatic MCF7 breast adenocarcinoma cells via *TP53/ATM*-mediated homologous recombination DNA double-strand break repair mediated through upregulation of *BRCA1* and downregulation of *RIF1*.

The upregulation of *UPF2* has been linked with impeded proliferation, G2/M cell cycle arrest, and migration defects in the knocked down ARA (Adriamycin; Doxorubicin Resistance Associated long non-coding RNA) in doxorubicin-resistant MCF7 cells [203]. Moreover, *UPF2* was associated with nonsense-mediated decay (NMD), which is a quality control mediated degradation of faulty transcripts [204,205] via the exon-junction complex (EJC) [206]. Interestingly, patients with brain metastatic breast cancer showed significant downregulation of the regulator of nonsense transcripts 2 protein (*UPF2*-encoded) in the brain metastases compared to the primary breast tumour [207]. Our study observed that the regulator of nonsense transcripts 2 was overexpressed in the synergistic combination-treated MCF7 cells. Thus, the *UPF2* overexpression by the synergistic AP-1 and DOX combination might be advantageous to overcome doxorubicin resistance in breast cancer cells with potential suppression of its metastasis.

Protein S100-A14, a member of EF-hand calcium-binding proteins, was the most downregulated protein in the synergistic combination-treated MCF7 cells (Table 2, Figure 9C). *HER2* is overexpressed in 20–25% of breast cancer, and it stimulates tumorigenesis through signalling molecules such as PI3K/AKT and MAPK/ERK [208]. S100-A14 protein is a modulator of HER2 signalling by directly binding to HER2 protein [208]. Previously, reduced HER2-stimulated cell proliferation was observed after silencing the *S100A14* in MCF7, BT474, and SK-BR3 breast cancer cells [208]. In addition, co-overexpression of *S100A14* with *S100A16* promoted the invasiveness of the MCF7 and SK-BR-3 breast adenocarcinoma cells [209]. Collectively, in our study, the synergistic combination of AP-1 and DOX reduced the expression of S100-A14, which in turn may have reduced the HER-2 stimulated proliferation and invasiveness of the MCF7 cells.

## 3. Conclusions and Future Directions

Strong synergistic interactions were observed between AP-1 and DOX against MCF7 cells using different synergy quantitation models with a promising CSS. Interestingly, CSS and S scores were able to capture the combination efficacy and synergy, respectively, for both CI-model data and its collective combination when reanalysed in DrugComb, unlike other synergy metrics (ZIP, LOEWE, BLISS, and HSA). However, ZIP, LOEWE, BLISS, and HSA synergy metrics were strongly correlated with CI values at different inhibitory concentrations.

Our results demonstrated that ROS depletion is associated with the MCF7 cell death after mono or combination treatment in a dose-dependent manner. Furthermore, the most synergistic combination led to a significant decline in ROS production in the MCF7 cells compared to monotherapy with DOX. However, the statistically significant ROS decline upon DOX treatment in the MCF7 cells in our study was not in agreement with the established DOX-induced ROS in cancers and normal tissue in the literature. Therefore, further studies using different doses of DOX with different treatment time points with the help of multiple ROS quantifying protocols and molecular studies are warranted to investigate any biphasic dose- and/or time-dependent DOX-mediated ROS production. In particular, the differential effects of antioxidants on DOX-mediated apoptosis and caspase 3 activity in normal vs. tumour cells reported in the literature support the use of AP-1 in combination with DOX. The observed ROS decline by DOX and/or AP-1 in the MCF7 cells along with the displayed apoptosis in our study suggests the involvement of other mechanisms controlling MCF7 cell death, highlighting the need for further mechanistic studies particularly amid the paradoxical and complex role of ROS in cancer.

AP-1 potentiated the anticancer activity of DOX by promoting apoptosis and facilitated a necrosis reversal to programmed cell death, which may be advantageous to decline DOX-related side effects. The observed necrotic to an apoptotic shift of DOX by the synergistic combination may be attributed to the antioxidant profile of AP-1 and the resultant antioxidant-related apoptotic pathways in the MCF7 cells. Nonetheless, further studies will be needed to completely characterise the underlying mechanisms of the observed necrosis to apoptosis shift by implementing a number of necrotic and apoptotic markers.

The enhanced apoptosis may be associated with upregulated expressions of pro-apoptotic p27, PON2, and catalase alongside downregulated anti-apoptotic XIAP, HSP60, and HIF-1α proteins. The AP-1 mediated overexpression of antioxidant proteins such as PON2 and catalase in the combination treatment group may be associated with the increased apoptosis of MCF7 cells and impeded oxidative stress-related side effects of DOX. In addition, the upregulated HTRA2/Omi, FADD, DR5, and DR4 contributed to the cytotoxic mechanisms of AP-1 against the MCF7 cells.

Label-free quantification-driven proteomics highlighted the top 21 differentially expressed proteins of MCF7 cells in the combination treatment group among the total identified 1687 proteins. AP-1 significantly synergised doxorubicin against the metastatic MCF7 breast adenocarcinoma cells via TP53/ATM-mediated homologous recombination for the DNA DSBs repair through *BRCA1* upregulation and *RIF1* downregulation. Undermined *HER-2* stimulated proliferation and invasiveness of the MCF7 cells may be expected due to the impeded expression of S100-A14 in the combination treatment versus the mono treatments. The enhanced expression of the *UPF2*-encoded regulator of nonsense transcripts 2 protein in the combination treatment group might be advantageous to overcome doxorubicin resistance in breast cancer cells.

We highlighted the prenylated stilbenes, flavanone, chalcone as potential anticancer metabolites of AP-1 against the MCF7 cells. Finally, AP-1 and its charted metabolites presented a new opportunity to enhance the effectiveness of the breast cancer treatment regimen containing DOX. However, further in vivo and clinical studies are warranted to validate these in vitro findings.

## 4. Material and Methods

### 4.1. Chemicals and Preparation of Australian Propolis Extract

Doxorubicin (DOX) with 98% purity was purchased from Sigma-Aldrich, New South Wales, Australia. In our recent study, we chemically standardised and identified the key markers of the ethanolic extract of Australian propolis (AP-1) [49]. The same AP-1 extract was utilised in the current study, where the previous study reported the extraction procedure [49].

### 4.2. Cell Culture

The MCF7 human breast adenocarcinoma and fibrocystic breast tissue MCF10A were obtained from the American Type Culture Collection (ATCC: Manassas, VA, USA). Dulbecco’s Modified Eagle Medium (DMEM; Lonza, New South Wales, Australia) with 4.5 g/L Glucose, L-Glutamine and sodium pyruvate (Lonza Australia Pty Ltd., Mount Waverley, Victoria, Australia) supplemented with 10% foetal bovine serum (FBS; Interpath, Victoria, Australia) and 100 U/mL of penicillin and streptomycin (Sigma-Aldrich, New South Wales, Australia) was used to culture the MCF7 at 37 °C in the presence of 5% CO_2_. The RAW 264.7 murine macrophage cell line was cultured using the same conditions except for 5% FBS in DMEM. DMEM/F12 supplemented with 20 ng/mL EGF, Caisson DFP18-1LT, 100 ng/mL cholera toxin, 5% horse serum, 0.5 μg/mL hydrocortisone, and 10 μg/mL insulin was used to culture the MCF10A cell line at 37 °C in the presence of 5% CO_2_. Viable cells were routinely quantified using the trypan blue exclusion assay with a cell counter (Vi-Cell XR Counter, Beckman Coulter GmbH, Krefeld, Germany).

### 4.3. Cell Viability Determination

Cellular viability was determined using the alamarBlue (resazurin) assay [210,211]. Briefly, in a 96 well plate, 100 μL of suspended MCF7 cells were seeded at 1 × 10^4^/well and incubated at 37 °C in the presence of 5% CO2 overnight to adhere. The cells were treated with different concentrations of AP-1 and DOX and their combinations in different ratios together with the vehicle control (0.5% dimethyl sulfoxide (DMSO)). After 72 h, the medium was removed from the wells, and 100 μL of working alamarBlue (0.1 mg/mL) solution was added to each well and incubated for 4 h at 37 °C in the presence of 5% CO_2_. The working alamarBlue solution was prepared by 1:10 dilution of freshly prepared stock (1 mg/mL resazurin in phosphate buffer saline) using FBS free media. Using a microplate reader (BMG CLARIOstar, Victoria, Australia), the fluorescence was measured with excitation wavelength at 555 nm and emission wavelength at 595 nm. Cell viability was determined as a percentage of the vehicle control.

### 4.4. Biochemometric and LCMS-Driven Metabolomic Identification of Anticancer Metabolites of AP-1 in the MCF7 Human Breast Adenocarcinoma Cells

AP-1 was fractionated using a preparative HPLC Shimadzu system (LC20AP Prep-pumps, SPD-20A Prominence UV/Vis detector, SIL-20A HT autosampler with FRC-10A fraction collector). Luna^®^ 5 µm C18 100 A°, LC column (250 mm × 21.2 mm) was utilised (Phenomenex, Torrance, CA, USA). Water and acetonitrile were used as mobile phase A and B, respectively. Gradient incline of acetonitrile at a flow rate of 15 mL min^−1^ was implemented with an initial 20% B, then the following gradient was used; at 25–20 min (40–60%B), 75–100 min (70–80% B), and 125–150 min (90–100% B) and washed for 10 min and equilibrated at 20% B for another 10 min. Two mL samples (250 µg mL^−1^ in acetonitrile) were injected, and five fractions were collected at 25 min intervals (Figure S7). The AP-1 fractions were dried and evaluated for their anticancer activity against MCF7. The fractions were analysed using ultra-high-performance liquid chromatography (UPLC) coupled with a quadrupole time of flight (qTOF) analyser using Acquity UPLC (Waters, Milford, MA, USA) coupled with SYNAPT G2-S (Waters, Milford, MA, USA) mass spectrophotometer. Five µL of the fractions (1 mg mL^−1^ in acetonitrile) was injected at 400 µL min^−1^. Chromatographic separation was achieved using ACQUITY UPLC HSS T3 Column (1.8 μm, 2.1 mm × 150 mm; Waters Corporation, Milford, MA, USA). The column temperature was kept at 45 °C, and gradient elution was implemented utilizing 0.1% formic acid solution of both water (A) and acetonitrile (B). Initially, 20% of the mobile phase B was used, and linearly inclined as the following gradient: 30–40% B (5-15 min), 40–60% B (15–20 min), and 60–90% B (20–28 min) and finally declined to 20% B for 30 min. G2-S high definition mass spectrometer (HDMS) (Waters Corp, Manchester, UK) equipped with Z-spray source controlled by MassLynx v4.1 was used for mass spectrometry analysis in negative ESI ionization mode using HDMS mode of operation. The scanning mode parameters were: source temperature: 120 °C, desolvation temperature: 500 °C, cone gas flow: 50 L/h, desolvation gas flow: 1000 L/h, collision energy ramp: 20–50 eV, capillary voltage: 2.5kV, and acquisition mass range: 50–1200 *m*/*z*.

Data were acquired in a profile mode and corrected with lock mass spray switching between the samples and external reference, allowing the MassLynx to ensure mass analysis accuracy continuously [212,213]. Leucine enkephaline (1 ng μL^−1^) was used as an external reference in 1:1 acetonitrile-water containing 0.1% formic acid at a flow rate of 5 μL/min via a LockSpray interface, generating a reference ion for negative ion mode [M-H]^−^ of 554.261 *m*/*z*.

Progenesis QI software (Waters Corp., Milford, MA, USA) was used for data processing, and features were considered reproducible if their coefficient of variation (CV) among the samples were < 25%, and the fold change (FC) > 2, ANOVA *p*-value and Q value < 0.01 against the blank samples. Orthogonal partial least-squares discriminant analyses (OPLS-DA) analyses were implemented to identify the discriminatory metabolites in the active fractions against inactive or less active ones using SIMCA version 14.1 (Umetrics, Umea, Sweden). Progenesis QI was used for putative identification of metabolites of interest by comparison with metabolomic profiling CCS library, LipidBlast, and Progenesis Metascape imported databases including HMDB, MONA, LipidMaps and GNPS and Chemspider imported data sources such as KEGG, NIST, in addition to reference literature and CRC dictionary of natural products database.

### 4.5. Synergy Quantification of AP-1 and DOX Combinations against the MCF7 Human Breast Adenocarcinoma Cells

The potential interactions between AP-1 and DOX were analysed using the combination index (CI) model and the DrugComb portal (https://drugcomb.fimm.fi/, accessed on 25 May 2021). CompuSyn version 2.0 (Biosoft, US) was used for the CI calculations based on the median-effect equation, which was derived from mass action law [214,215,216]. In the current study, nine pairwise combinations of DOX with AP-1 were studied in constant ratio design with a six-points dose-response curve in 2:1 serial dilution (*n* = 3) using the CI model. Furthermore, the combinations were also evaluated in a checkerboard design (*n* = 3) using drugComb [217]. The response data obtained from the CI model were further analysed in DrugComb, where the mean percentage of cell inhibition and the concentrations of the combined drugs were used as input for synergy scores in different models and combination sensitivity score (CSS) evaluation.

### 4.6. Reactive Oxygen Species (ROS) Assay

The intracellular ROS level in the MCF7 cells treated with AP-1, DOX and their most synergistic combination was evaluated using the DCFDA (2′,7′-dichlorofluorescein diacetate) Cellular ROS Detection Assay Kit (#ab113851; Abcam, Victoria, Australia) according to the manufacturer’s protocol. DCFDA is a fluorogenic dye that measures hydroxyl, peroxyl and other ROS activity within the cell. Briefly, in a 96 well-plate, the MCF7 cells were seeded at 2.5 × 10^4^ cells/well and incubated at 37 °C in the presence of 5% CO_2_ overnight to adhere. The next day, the media was discarded, and cells were washed with 100 µL/well of the 1X buffer. Then the cells were incubated at 37 °C with 100 µL per well of 20 µM DCFDA solution for 45 min in the dark. Then, the DCFDA solution was discarded, and cells were washed with 100 µL per well of 1X buffer and treated with different concentrations of AP-1, DOXO, synergistic combinations, and positive control tert-Butyl hydroperoxide (TBHP) for 4 h. The plate was measured immediately at Ex/Em = 485/535 nm in endpoint by using a microplate spectrophotometer (BMG CLARIOstar, Victoria, Australia). The assay protocol is based on the diffusion of DCFDA into the cell, which is then deacetylated by cellular esterases to a non-fluorescent compound, which is then ROS oxidised into 2′,7′–dichlorofluorescein (DCF) that can be detected using a fluorescence plate reader. Blank readings for treatments were subtracted, and the percentage of ROS production was calculated relative to the negative control (no treatment).

### 4.7. Flow Cytometric Analyses of Apoptosis in the MCF7 Human Breast Adenocarcinoma Cells Using Annexin V-CF Blue and 7-Aminoactinomycin D (7AAD)

The apoptotic profiles of the MCF7 human breast adenocarcinoma cells after treatment with AP-1, DOX and their most synergistic combination were analysed using the Abcam Apoptosis Detection Kit (#ab214663, Abcam, Victoria, Australia) as per the manufacturer’s protocol. Briefly, the MCF7 cells were cultured in T75 cell culture flasks with a seeding density of 1 × 10^6^ and exposed to vehicle control (DMSO), AP-1 (100 µg mL^−1^), DOX (0.29 µg mL^−1^) and the synergistic combinations of AP-1 and DOX (50 µg mL^−1^: 0.145 µg mL^−1^). After 24 h, the cell culture media was collected, and each cell flask was treated with 0.25% *w*/*v* of trypsin for 3 min at 37 °C. Trypsin was neutralised with an equal volume of 10% FBS-containing media and combined with the previously collected media. Cell pellets were collected by centrifugation at 500× *g* for 5 min at room temperature (RT), washed twice in PBS, resuspended in 1 mL PBS, and centrifuged at 500× *g* for another 5 min. Harvested cell pellets of all treatment groups were immediately resuspended in 0.5 mL 1× binding buffer, and to each 100 µL of cell suspension, 5 µL of annexin V-CF blue and 7-AAD staining solutions were added. Cells were incubated in dark at RT for 15 min, and then 400 µL of 1× binding buffer was added. The cells were then analysed by ACEA Biosciences Novocyte 3000 flow cytometer (ACEA Biosciences Inc., San Diego, CA, USA). The NovoExpress (ver 1.5.0, ACEA Biosciences Inc., USA) software was implemented for analysis and processing where cells were gated on FSC vs. SSC to exclude the debris near the origin and cell aggregates. This was followed by gating on dot-plots of Annexin V-CF in Pacific Blue vs. 7-AAD fluorescence in PerCP with a quadrant placed indicating live cells (+Annexin V and-7-AAD) in the lower-left quadrant, early apoptotic cells (+Annexin V and −7-AAD) in the lower-right quadrant, late apoptotic cells (+Annexin V and +7-AAD) in the upper-right quadrant and necrotic cells (−Annexin V and +7-AAD) in the upper-left quadrant. Finally, cell percentage data in each quadrant after different treatments (*n* = 4) were exported to GraphPad Prism (version 9.0, San Diego, CA, USA) for statistical analysis and visualisation.

### 4.8. Human Apoptosis Proteomic Array

#### 4.8.1. Cell Culture, Treatment, and Protein Extraction

The MCF7 cells were cultured in T75 cell culture flasks with a seeding density of 1.0 × 10^6^ cells and incubated overnight at 37 °C in the presence of 5% CO_2_. The media was aspirated and replaced with fresh media containing 0.5% DMSO as the vehicle control, 100 µg mL^−1^ AP-1, 0.29 µg mL^−1^ DOX and synergistic combination (100 µg mL^−1^ AP-1, and 0.29 µg mL^−1^ DOX), then incubated for 24 h at 37 °C in the presence of 5% CO_2_. The cell culture media was collected, and each cell flask was treated with 0.25% *w/v* trypsin for 3 min at 37 °C. Trypsin was neutralised with an equal volume of 10% FBS-containing media and combined with the previously collected media. The cells were centrifuged at 500× *g* for 5 min at RT, and the pellets were washed twice with ice-cold PBS and centrifuged again at 500× *g* for 5 min. The cell pellets were then resuspended in 100 µL lysis buffer included in proteome profiler human apoptosis array kit (ARY009, R&D Systems, NE Minneapolis, MN, USA). The lysis buffer was freshly supplemented with cOmplete Protease Inhibitor Cocktail™ (#04693116001; Roche UK purchased from Sigma-Aldrich, New South Wales, Australia). Cell pellets were left on ice for 20 min with occasional vortexing for 10 sec every 5 min, then centrifuged at 14,000 rpm for 20 min at 4 °C, and the lysate was collected.

#### 4.8.2. Protein Quantification

Pierce™ Rapid Gold BCA Protein Assay Kit (#A53226, ThermoScientific, Waltham, MA, USA) was used to determine the protein concentration of the cell lysate in triplicates against bovine serum albumin (BSA) standard according to the manufacturer’s protocol. Briefly, 1 µL of each sample replicate was 1:20 diluted in the water together with 20 µL of each standard, were placed in a 96-well plate with 200 µL of working reagent per well. Samples were diluted to be within the operating range of 20–2000 µg mL^−1^. The plate was mixed thoroughly on a plate shaker for 30 s and incubated at room temperature for 5 min, and then the absorbance was measured within 20 min at 480 nm using a microplate spectrophotometer (BMG CLARIOstar, Victoria, Australia). The blank absorbance was subtracted from all other readings of standards and samples, and sample concentration was determined against the established BSA standard calibration curve. Samples were stored at −80 °C for further analysis.

#### 4.8.3. Apoptosis Proteome Array Analysis

A proteome profiler^TM^ human apoptosis array kit (#ARY009, R&D Systems, NE Minneapolis, MN, USA) was used according to the manufacturer’s instructions to analyse the expression level of 35 apoptosis-related proteins in the MCF7 cell lysates treated with AP-1, DOX, their synergistic combination and the vehicle control. Briefly, each array was blocked (2 mL array buffer 1) for 1 h, then incubated with the MCF7 cell lysates (350 µg total protein) for 2 h at RT and washed three times (10 min each) on a rocking platform shaker. The arrays were mixed with antibody cocktails and incubated for 1 h, then washed and incubated for 30 min with Streptavidin-HRP. The arrays were rewashed three times (10 min each) and incubated with the Chemi Reagent mix for 1 min. The extra Chemi Reagent was wiped, and blot images were captured using an ImageQuant^TM^ LAS 500 image system (GE, Healthcare, Bio-Sciences, Uppsala, Sweden) with 2 min of manual exposure. The pixel densities of the developed spots were analysed using ImageJ [218], and mean negative control pixel intensities (PBS) were subtracted from all values followed by pairwise comparisons of the expression data of single treatments versus the control array (MCF7 cell treated with 0.5% DMSO) or the synergistic combination versus single treatments. All expression data were quantile normalised, log-transformed, and Pareto-scaled before any statistical analyses. Statistical analysis was performed using Metaboanalyst 5.0 [219] for the selection of significantly dysregulated proteins (Absolute fold change (FC) of 1.3 and *p*-value ≥ 0.05) after different treatments in pairwise comparisons.

### 4.9. Bottom-Up Label-Free Quantification Proteomic Study of the MCF7 Cell Lysates after Treatment with the Most Synergistic Combination

#### 4.9.1. Cell Culture, Treatment and Protein Extraction

The MCF7 cells were cultured in T75 flasks at a seeding density of 1.0 × 10^6^ cells and incubated overnight at 37 °C in the presence of 5% CO_2_. The media was aspirated and replaced with fresh media containing 0.5% DMSO as the vehicle control, 100 µg mL^−1^ AP-1, 0.29 µg mL^−1^ DOX and synergistic combination (100 µg mL^−1^ AP-1, and 0.29 µg mL^−1^ DOX), and incubated for 24 h at 37 °C in the presence of 5% CO_2_. The cell culture media was collected, and each cell flask was treated with 0.25% *w*/*v* trypsin for 3 min at 37 °C. Trypsin was neutralised with an equal volume of 10% FBS-containing media and combined with the previously collected media. The cells were centrifuged at 500× *g* for 5 min at RT, and the pellets were washed twice with ice-cold PBS and centrifuged again at 500× *g* for 5 min. The cell pellets were then resuspended in 100 µL lysis buffer with 1 µL of universal nuclease included in EasyPep™ Mini MS Sample Prep Kit (ThermoFisher Scientific, USA). Halt™ Protease and Phosphatase Inhibitor Cocktail, EDTA-Free (Thermo Scientific, USA) were used at 10 µL mL^−1^ of lysis buffer to prevent enzymatic protein degradation during extraction and purification protocols. This cocktail is fully compatible with Pierce cell lysis buffers can be used safely in mass spectrometry (MS). The cells were pipetted up and down 10–15 times until sample viscosity is reduced and left in ice for 20 min, then centrifuged at 14,000 rpm for 20 min at 4 °C, and the lysate was collected.

#### 4.9.2. Peptides Preparation and Clean Up

The cell lysates were quantified as in Section 4.8.2, and 100 µg of protein samples were used for chemical and enzymatic sample processing according to the manufacturer protocol (EasyPep™ Mini MS Sample Prep Kit; ThermoFisher Scientific, USA). The final volume was adjusted to 100 µL using lysis buffer in a microcentrifuge tube. 50 µL of the reduction and alkylation solutions were added, gently mixed, and incubated at 95 °C using a heat block for 10 min. The samples are allowed to cool at RT, then 50 µL of the reconstituted trypsin/lys-C protease mixture was added to each sample and incubated with shaking at 37 °C for 3 h. After incubation, 50 µL of digestion stop solution was added and mixed gently. Peptides clean up columns were implemented to remove hydrophilic and hydrophobic contaminants where clean peptide samples were dried using a vacuum centrifuge and resuspended in 100 µL 0.1% formic acid in water for LC-MS analysis.

#### 4.9.3. Label-Free Bottom-Up Quantification via Nano-Ultra High-Performance Liquid Chromatography Coupled with Quadruple Time of Flight Mass Spectrometry (NanoUPLC-qTOF-MS)

Tryptic peptides were analysed using a nanoACQUITY UPLC system (Waters Corp., Milford, MA, USA) coupled to Synapt G2-S high-definition mass spectrometer (HDMS) (Waters Corp., Manchester, UK) operating in positive electron spray ion mode (ESI+) and equipped with hybrid quadrupole time of flight (qTOF) analyser. Mass accuracy was maintained by Waters NanoLockSpray Exact Mass Ionization Source with 100 fg mL^−1^ Glu-fibrinopeptide B (GFP) Lockspray solution (in 50% aqueous acetonitrile containing 0.1% formic acid, lock mass *m*/*z* 785.84.26) infused at 0.5 μL min^−1^ and calibrated against a sodium iodide solution. The chromatographic system was equipped with a nanoEase M/Z BEH C18 (1.7 μm, 130 Å, 75 μm × 100 mm, Waters Corp., Milford, MA, USA) at 40 °C and nanoEase M/Z Symmetry C18 Trap Column (100 Å, 5 µm, 180 µm × 20 mm, Waters Corp., USA). Milli-Q water and acetonitrile (LCMS grade, Merck, Germany) containing 0.1% formic acid were used as mobile phase A and B, respectively, with 1 µL injection volume at 0.3 µL min^−1^ flow rate throughout 50 min gradient. Samples were injected into the trapping column at 5 μL min^−1^ at 99% mobile phase A for 3 min before being eluted on the analytical column. The peptides were separated using a chromatographic method where an initial 1% of mobile phase B and ramped to 85% B over 50 min with the following gradient: 10% B at 2 min, 40% B at 40 min and 85% B at 42 min. All samples are kept at 4 °C and were injected in triplicates. The ion source block temperature was set to 80 °C, and capillary voltage was maintained to 3 kV. Ions were acquired with *m*/*z* between 50 and 2000, scanning time of 0.5 sec, sample cone voltage and source offset at 30 V, nanoflow gas at 0.3 Bar, purge gas at 20 L h^−1^ and cone gas flow at 20 L h^−1^. Data independent acquisition (DIA) method by MS^E^ multiplex mode was used for samples acquisition at T-wave collision-induced dissociation cell filled with argon gas with MassLynx Mass Spectrometry Software (Waters Corporation, USA).

#### 4.9.4. Data Processing and Availability

Progenesis QI software (Waters Corporation, Milford, MA, USA) was used to import and further process the MassLynx acquired data. Automatic selection of alignment reference among QC samples was set, and peptides were identified against Uniprot human proteome database (October 2020 version) using the ion accounting method with 250 kDa protein mass maximum. One fragment per peptide or one peptide per protein together with three fragments per protein were set as ion matching requirements using relative quantification implementing the Hi-N method (*n* = 3). Auto peptide and fragment tolerance and less than 4% FDR were set for search tolerance parameters. Peptides with absolute mass error > 20 ppm or single charged were further filtered out. Pairwise comparisons of the identified proteins in the treated groups were done against the control group for potential cytotoxic exploration, while the most synergistic combination samples were compared against both DOX and AP-1 -treated samples to elucidate possible synergistic mechanisms.

In each experimental design, proteins with analysis of variance (ANOVA)-derived *p*-value ≤ 0.05 and q value ≤ 0.01 with absolute fold change (FC) ≥ 1.7 were considered significant and included for further pathway analyses. Differentially expressed proteins identified by the quantitative processing of the LC-MS/MS analysis of the proteome tryptic digestion were analysed by STRING [220], Reactome [221], g:Profiler [222,223] and IMPaLA [224] to identify the relevant pathways responsible for the synergistic effect against the MCF7 cells. The G:SCS algorithm was used for multiple testing corrections in g:profiler platform with an adjusted p-value of 0.05 threshold. The raw and processed data have been deposited to the ProteomeXchange Consortium via the PRoteomics IDEntifications (PRIDE) repository [225] with the dataset identifier PXD026331 and 10.6019/PXD026331.

### 4.10. Statistical Analysis

All statistic comparisons were performed using GraphPad Prism Version 9 (San Diego, CA, USA) except for the apoptotic array and shotgun proteomics study where MetaboAnalyst 5.0 (https://www.metaboanalyst.ca/, accessed on 2 June 2021) were used together with Progenesis QIP (Waters Corporation, Milford, MA, USA) for the shotgun proteomic study. The significance was analysed by ANOVA and *t*-test for multiple and pairwise comparisons, respectively. Data were expressed as a mean ± SD. The differences between the mean values in the experiments at least *p* < 0.05 were considered statistically significant.

## Figures and Tables

**Table 1:** Synergy quantitation of AP-1 and DOX combinations against the MCF7 breast adenocarcinoma cells.

Combo ID	Highest Dose (µg mL^−1^)		IC_50_ ± SD	Ratio (*w*/*w*)	CI Values at:					CSS	S	ZIP	BLISS	LOEWE	HSA
	AP-1	DOX	(µg mL^−1^)		ED_50_	ED_75_	ED_90_	ED_95_	ED_97_						
Propolis			62.95 ± 9.28												
Doxorubicin			0.24 ± 0.03												
PDOX19	20	0.52	62.95 ± 9.28	100:2.61	1.67	1.3	1.03	0.88	0.79	75.38	44.86	−10.43	−12.09	−5.89	−2.22
PDOX28	40	0.46	11.25 ± 4.59	100:1.16	2.5	2.23	2.05	1.95	1.9	72.4	41.07	−14.33	−16.63	−10.69	−6.43
PDOX37	60	0.41	28.10 ± 4.32	100:0.68	2.93	2.75	2.69	2.7	2.72	73.35	40.57	−15.11	−19.6	−13.43	−8.88
PDOX46	80	0.35	40.55 ± 4.22	100:0.43	1.96	2.72	2.75	3.2	3.59	77.25	43.88	−13.51	−16.12	−9.71	−5.03
PDOX55	100	0.29	49.03 ± 16.99	100:0.29	0.77	**0.57**	**0.44**	**0.38**	**0.35**	84.41	**51.85**	−6.69	−8.52	−2.82	**1.78**
PDOX64	120	0.23	36.93 ± 15.24	100:0.19	0.75	**0.64**	**0.58**	**0.55**	**0.54**	84.81	**51.87**	−8.05	−9.62	−3.52	**1.55**
PDOX73	140	0.17	41.88 ± 18.87	100:0.12	0.98	0.88	0.83	0.82	0.82	82.35	49.75	−9.64	−11.25	−4.93	−0.21
PDOX82	160	0.12	54.97 ± 7.04	100:0.07	0.98	0.82	**0.71**	**0.66**	**0.63**	83.27	**51.94**	−6.66	−8.04	−1.59	**2.49**
PDOX91	180	0.06	58.48 ± 3.10	100:0.03	1.24	1.34	1.4	1.47	1.64	80.54	49.78	−6.05	−7.95	−1.31	**1.98**
**CI to DC**										80.74	**55.69**	−9.05	−10.59	−6.46	−2.17
**Checkerboard**										65.39	32.65	**4.5**	**4.49**	0.96	**4.28**
Selected Dose *					0.11 (94% inhibition) ^#^										
	➢ CI to DC											**22.55**	**16.65**	**9.95**	**40.08**
	➢ Checkerboard design											−1.27	0.25	**4.52**	**10.05**

BLISS; Bliss independence synergy model; CI = Combination index model; CI to DC = all combined responses from CI model combinations were analysed via the DrugComb server; CSS; Combination sensitivity score; HSA = highest single agent model; LOEWE; Loewe additivity synergy model; S; Synergy model derived from CSS; ZIP = zero interaction potency model; * = 100 µg mL^−1^ AP-1 and 0.29 µg mL^−1^ DOX; ^#^ = 88% inhibition was indicated in checkerboard assay. Potential synergistic combinations with CI < 0.75 or synergy score > 1.5 in Loewe, HSA, ZIP, Bliss models or > 50 in S synergy model were bold formatted.

**Table 2:** Differentially expressed proteins in the MCF7 cells treated with the synergistic AP-1 and DOX combination.

Uniprot Accession	Gene	Fold Change	Description
Upregulated Proteins			
H0YMP8	*ATP8B4*	15.05	Phospholipid-transporting ATPase
A0A0A0MQS9;Q16363	*LAMA4*	4.60	Laminin subunit alpha-4
Q9HAU5	*UPF2*	3.22	Regulator of nonsense transcripts 2
Q9Y216	*SEC23A*	2.53	Myotubularin-related protein 7
P38398	*BRCA1*	1.77	Breast cancer type 1 susceptibility protein
E9PDF1	*SH3TC2*	1.74	SH3 domain and tetratricopeptide repeats 2
Downregulated Proteins			
Q9HCY8	*S100A14*	18.96	Protein S100-A14
H3BUX2	*CYB5B*	5.09	Cytochrome b5 type B
Q8N5U6	*RNF10*	3.73	RING finger protein 10
H7C2B5	*RIF1*	3.58	Telomere-associated protein RIF1
Q8N9V7	*TOPAZ1*	2.86	Protein TOPAZ1
Q14676	*MDC1*	2.63	Mediator of DNA damage checkpoint protein 1
Q12873	*CHD3*	2.59	Chromodomain-helicase-DNA-binding protein 3
Q9NSB2	*KRT84*	2.49	Keratin_ type II cuticular Hb4
A0A087WWY9; Q8TB24	*RIN3*	2.35	Ras and Rab interactor 3
P62913	*RPL11*	2.32	60S ribosomal protein L11
D6RCP5	*ARHGAP24*	2.08	Rho GTPase-activating protein 24
Q9NYL9	*TMOD3*	1.88	Tropomodulin-3
Q9Y2H9	*MAST1*	1.83	Microtubule-associated serine/threonine-protein kinase 1
Q6ZRV2	*FAM83H*	1.82	Protein FAM83H
P14854	*COX6B1*	1.77	Cytochrome c oxidase subunit 6B1

AP-1 = Australian propolis extract; DOX = Doxorubicin

**Table 3:** Significantly overrepresented pathways identified via Reactome, STRING and g:Profiler using differentially expressed protein in the MCF7 cells treated with the synergistic AP-1 and DOX combination.

Platform.	Process/Pathway	*p*-Value	FDR	Present Entities
Reactome	TP53 Regulates Transcription of DNA Repair Genes	1.78 × 10^−8^	3.29 × 10^−06^	*MDC1*; *BRCA1*
	Transcriptional Regulation by TP53	2.72 × 10^−06^	2.50 × 10^−04^	*MDC1*; *BRCA1*; *CHD3*; *COX6B1*
	NHEJ	1.84 × 10^−04^	1.12 × 10^−02^	*MDC1*, *RIF1*, *BRCA1*
	G_2_/M DNA damage checkpoint	1.07 × 10^−02^	1.07 × 10^−01^	*MDC1*; *BRCA1*
	Cell Cycle Checkpoints	1.64 × 10^−02^	1.47 × 10^−01^	*MDC1*; *MAST1*; *BRCA1*
	Nonsense-Mediated Decay (NMD)	2.38 × 10^−02^	1.67 × 10^−01^	*UPF2*; *RPL11*
STRING	DSBs repair via NHEJ	NA	0.047	*MDC1*, *RIF1*, *BRCA1*
	BRCT, breast cancer carboxy-terminal domain	NA	0.022	*MDC1*, *BRCA1*
g:Profiler	NHEJ	1.30 × 10^−2^	NA	*MDC1*, *RIF1*, *BRCA1*
	DNA IR-DSBs and cellular response via ATM	7.00 × 10^−3^	NA	*MDC1*, *RIF1*, *BRCA1*

DSB; double-strand breaks, NHEJ; Nonhomologous End-Joining, underlined values are >0.05. Red and blue are up- and downregulated entities, respectively.

## Data Availability

Shotgun proteomics data is available in the PRIDE repository with the dataset identifier PXD026331 and DOI; 10.6019/PXD026331. All other data are presented within the article or Supplementary Files 1 and 2.

## Supplementary Materials

The following are available online at https://www.mdpi.com/article/10.3390/ijms22157840/s1.

## Abbreviations

7-AAD	7-aminoactinomycin D
AC	Adriamycin/cyclophosphamide
AP-1	Australian propolis sample 1
Apaf-1	Apoptotic protease-activating factor 1
ARA	Adriamycin Resistance Associated long non-coding RNA
ATM	Ataxia-telangiectasia mutated
ATR	Ataxia-telangiectasia and Rad3-related protein
Bad	Bcl-2 associated agonist of cell death
Bax	BCL2 associated X
Bcl-2	B-cell lymphoma 2
Bcl-XL	B-cell lymphoma-extra large
CMF	cyclophosphamide/methotrextate/5-flurouricil
CAT	cyclophosphamide/adriamycin/taxanes
CI	Combination index
cIAP-1	Cellular Inhibitor of Apoptosis Protein 1/Baculoviral IAP repeat-containing 2
cIAP-2	Baculoviral IAP repeat containing 3
CSS	Combination sensitivity scores
DSBs	Double-strand breaks
DOX	Doxorubicin
EJC	Exon-junction complex
ER+	Estrogen receptor positive
FADD	Fas-associated protein with death domain
Fas/TNFRSF6/CD95	Fas receptor/tumour necrosis factor receptor superfamily member 6/cluster of differentiation 95
HER-2	Human epidermal growth factor receptor 2/Receptor tyrosine-protein kinase erbB-2
HIF-1α	Hypoxia-inducible factor 1-alpha
HO-1/HMOX1/HSP32	Heme oxygenase 1
HO-2/HMOX2	Heme oxygenase 2
HSP27	Heat shock protein 27
HSP60	Heat shock protein 60 chaperonins
HSP70	Heat shock protein 70
HTRA2/Omi	High-temperature requirement protein A
JNK	c-Jun N-terminal kinase
IAPs	Inhibitors of apoptosis
IGF-1	Insulin-like growth factor-1
NHEJ	Nonhomologous End-Joining
NMD	Nonsense-mediated decay
ROS	Reactive oxygen species
S	S synergy score
VIP	Variable Importance Projection score derived from PLS-Da model
TRAIL R1/DR4	TNF-related apoptosis-inducing ligand receptor 1/Death receptor 4
TRAIL R2/DR5	TNF-related apoptosis-inducing ligand receptor 2/Death receptor 5
PON2	Serum paraoxonase/arylesterase 2
p21/CIP1/CDKN1A	cyclin-dependent kinase inhibitor 1
p27/Kip1	Cyclin-dependent kinase inhibitor 1B
TNF RI/TNFRSF1A	Tumour necrosis factor receptor 1/tumour necrosis factor receptor superfamily member 1A
XIAP	X-linked inhibitor of apoptosis protein
