# Supplementary material for: Metabolomic Identification of Anticancer Metabolites of Australian Propolis and Proteomic Elucidation of Its Synergistic Mechanisms with Doxorubicin in the MCF7 Cells
Source: Int J Mol Sci. 2021 Jul 22;22(15):7840. doi: 10.3390/ijms22157840 (PMC8346082; doi:10.3390/ijms22157840)
Supplement: Supplementary file 1 [file ijms-22-07840-s001.zip › Supplementary files/Supplementary file 1.pdf]

**Metabolomic identification of anticancer metabolites of Australian propolis and proteomic elucidation of its synergistic mechanisms with Doxorubicin in the MCF7 cells**

Muhammad A Alsherbiny[1,2], Deep Jyoti Bhuyan[1], Ibrahim Radwan[3], Dennis Chang[1], Chun Guang Li[1]

[1]NICM Health Research Institute, Western Sydney University, Penrith, NSW, Australia

[2]Department of Pharmacognosy, Faculty of Pharmacy, Cairo University, Cairo, 11562, Egypt

[3]Faculty of Science and Technology, University of Canberra, ACT, Australia

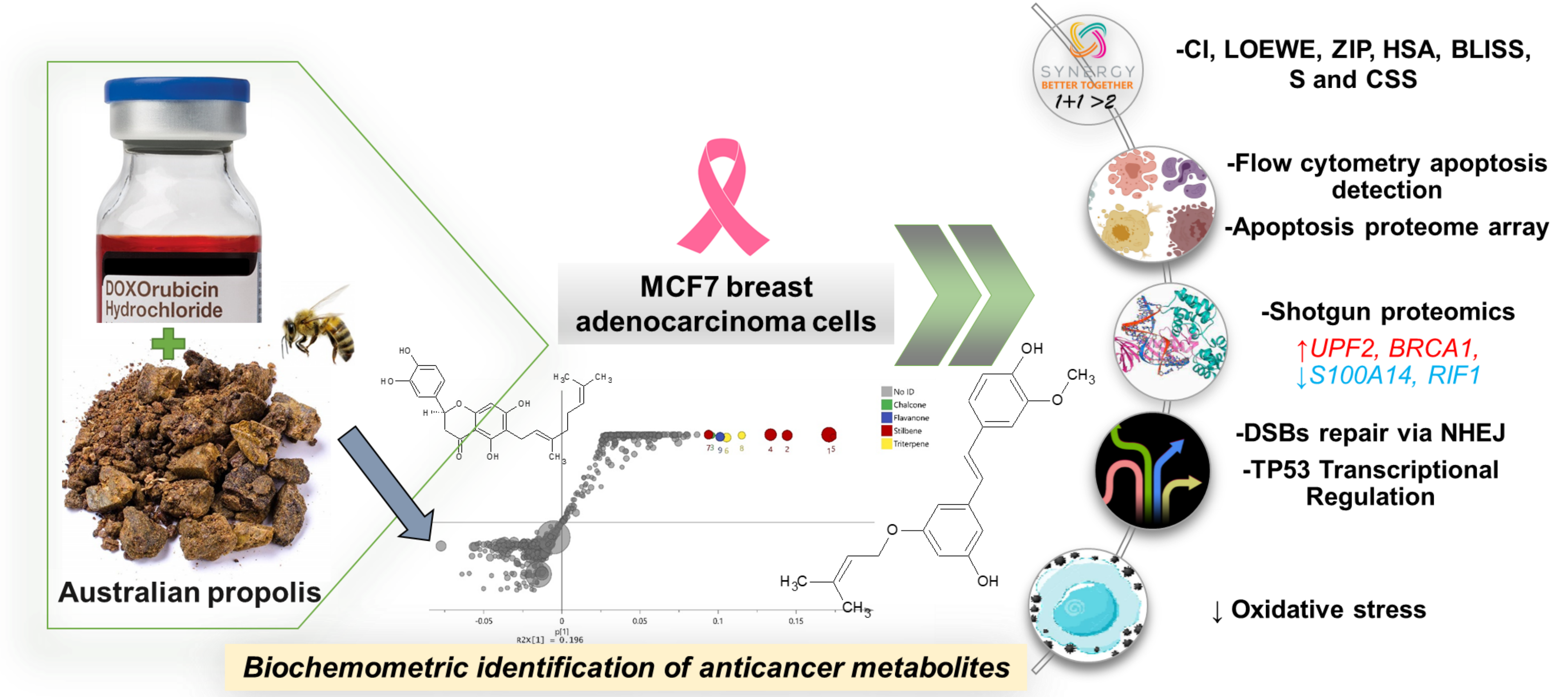

## List of Supplementary tables

Table S1 Metabolomic-driven identified active metabolites of propolis samples against MCF-7 cells using UPLC-qTOF-MS and chemometrics ........ 4
Table S2 Pearson's correlation of different DrugComb metrics and CI values at different in inhibitory concentration ........ 5
Table S3 Percentage of necrotic, apoptotic, and living cells retrieved from Annexing V-7AAD flowcytometry apoptosis analysis among propolis, doxorubicin and their mixture ........ 5
Table S4 Human apoptosis array coordinates and corresponding proteins ........ 6
Table S5 Significant apoptotic proteins in MCF-7 cell lysate upon treatment by doxorubicin, propolis or its synergistic combination.. 7
Table S6 Reactome overrepresented pathways using dysregulated proteins in MCF-7 cells treated with the synergistic propolis and doxorubicin combination ........ 9
Table S7 IMPala overrepresented pathways using dysregulated proteins in MCF-7 cells treated with the synergistic propolis and doxorubicin combination ........ 10

## List of supplementary figures

Figure S1 Viability of RAW 264.7 and MCF10A cells upon treatment with AP-1 and PDOX55 along the dose-response curve of DOX against MCF10A cells. ........ 11
Figure S2 Dose-response curves of (A) Australian propolis (Prop), doxorubicin (Dox) and most synergistic combinations (B) together with its combination index (CI) plot (C) calculated from CompuSyn software. ........ 12
Figure S3 Emission spectra for Annexin V and 7-AAD using PerCP and Pacific blue channels (https://www.biolegend.com/en-us/spectra-analyzer) ........ 13
Figure S4 Combined cell percentage analysis in different treatment groups in quadruplicates. Twenty-four hours treatment of propolis extract (100 $\mu gmL^{-1}$), Dox; doxorubicin (0.22 $\mu gmL^{-1}$), and their mixture at a half-dose synergistic combination (50 $\mu gmL^{-1}$ and 0.11 $\mu gmL^{-1}$, respectively) with a negative control were implemented using antibodies against Annexin-V CF-Blue and the reporter 7AAD. ****; significantly different as derived from Two-way ANOVA and Tukey's multiple comparisons at $P<0.001$, n=4. . 13
Figure S5 Supervised PLS-DA and Unsupervised PCA multivariate data analysis of the apoptotic proteins of MCF-7 lysates analysed by Proteome profiler human apoptotic array kit. (A) Score plot of principal component analysis (PCA) between the selected principal components alongside the explained variances shown in brackets. (B) Score plot of partial least square discriminant analysis (PLS-DA) where third averaged point was imputed for each treatment, (C and D) are the most discriminatory proteins in among different

treatments identified by variable importance projection (VIP) score and coefficients of PLS-Da model, respectively. The coloured boxes on the right indicate the relative concentrations of the corresponding metabolite in each group under study. ........ 14
Figure S6 Reactome downloaded TP53 transcription regulation pathway (A), TP53 transcriptional regulation of DNA repair genes with the enriched genes of BRCA1 and MCD1 marked by an orange star. ........ 15
Figure S7 Preparative HPLC fractionation of Australian propolis extract ........ 17

**Table S1 Metabolomic-driven identified active metabolites of propolis samples against MCF-7 cells using UPLC-qTOF-MS and chemometrics**

| CPD ID | m/z | Rt (min) | CCS ($A^2$) | Accepted Identification | Adducts* | Formula | Class | $MS^n$ | Mass Error (ppm) |
|---|---|---|---|---|---|---|---|---|---|
| 1 | 341.1358 | 16.71 | 181.5785 | Prenyl-tertrahydroxy-methoxy-stilbene (5,2',3',4'-tetrahydroxy-3-methoxy-2-prenyl-(E)-stilbene) | M-H | C20H22O5 | Stilbene | **341.1419(100%),** 323.1281, 244.0374, 242.0561, **188.0480**, 144.0580 | -10.58 |
| 2 | 341.1374 | 17.29 | 181.5784 | Prenyl-tetrahydroxy-methoxy-stilbene (5,6,3',4'-tetrathydroxy-3-methoxy-2-prenyl-(E)-stilbene) | M-H | C20H22O5 | Stilbene | 341.1406, 323.1273, 244.0374, 242.0571, **188.0479 (100%),** 144.0579 | -5.99 |
| 3 | 323.1264 | 18.17 | 183.7832 | Prenyl-trihydroxy-chalcone | M-H | C20H20O4 | Chalcone | 323.1281(100%), 255.0615 254.0567, 226.0616 | -7.63 |
| 4 | 325.1337 | 18.22 | 181.9172 | Trihydroxy-methoxy-prenyl-(E)-stilbene | M-H | C20H22O4 | Stilbene | 325.1468, 272.0685, 241.0497, **188.0491 (100%),** 144.0583 | -14.74 |
| 5 | 325.1417 | 18.79 | 180.0948 | Dihydroxy-methoxy-prenyloxy-(E)-stilbene | **M-H**, M-H2O-H | C20H22O4 | Stilbene | 325.1477, 241.0503, **188.0499 (100%),** 144.0587 | -8.61 |
| 6 | 489.3525 | 19.00 | 229.2606 | Undefined triterpene | M-H2O-H, **M-H** | C30H50O5 | Triterpene | M-H (489.3601 (100%), 235.1326, 207.1019, M-H2O-H (471.3428,235.1327, 207.1020, 144.9221) | -14.72 |
| 7 | 379.1881 | 19.14 | 213.2551 | Bisprenyl-tetrahydroxystilbene [(E)-2,6-bis(3-methyl-2-buten-1-yl)-3,3',5,5'-tetrahydroxystilbene] | M-H2O-H, **M-H**, M+FA-H | C24H28O4 | Stilbene | 379.1935 (100%), 309.1111, 323.1289, 267.0652, 255.0663, 254.0558, 257.1533 | -8.92 |
| 8 | 489.3535 | 19.68 | 229.2606 | Undefined triterpene | M-H | C30H50O5 | Triterpene | 489.3598(100%), 235.1329, 207.1017 | -10.23 |
| 9 | 423.1806 | 20.94 | 210.7108 | Geranyl-tetrahydroxyflavanone (propolin C/D/F) | M-H2O-H, **M-H**, M+Na-2H, M+K-2H | C25H28O6 | Flavanone | 423.1822 (M-H), 287.1306 (100%), 135.0472 (95%) | -8.66 |

CCS; collision cross-section, *; Bold adducts are the most abundant ones with the reported precursor ion m/z and CCS.

**Table S2 Pearson's correlation of different DrugComb metrics and CI values at different in inhibitory concentration**

| | CI at | | | | |
|---|---|---|---|---|---|
| | ED50 | ED75 | ED90 | ED95 | ED97 |
| CSS | 0.96 | 0.89 | 0.83 | 0.78 | 0.75 |
| S | 0.95 | 0.88 | 0.83 | 0.78 | 0.75 |
| ZIP | 0.83 | 0.78 | 0.74 | 0.71 | 0.68 |
| BLISS | 0.83 | 0.78 | 0.74 | 0.71 | 0.68 |
| LOEWE | 0.81 | 0.75 | 0.70 | 0.67 | 0.64 |
| HSA | 0.87 | 0.81 | 0.76 | 0.72 | 0.69 |

**Table S3 Percentage of necrotic, apoptotic, and living cells retrieved from Annexing V-7AAD flowcytometry apoptosis analysis among propolis, doxorubicin and their mixture**

| | **% Necrotic cells** | **% late apoptotic cells** | **%living cells** | **% Early apoptotic cells** |
|---|---|---|---|---|
| **Negative Control** | $0.56\pm0.32^{a}$ | $8.7\pm2.39^{a}$ | $87.63\pm2.52^{a}$ | $3.12\pm0.35^{a}$ |
| **Propolis** | $3.41\pm0.842^{a}$ | $43.53\pm12.89^{b}$ | $10.04\pm8.22^{b}$ | $43.02\pm5.46^{b}$ |
| **Doxorubicin** | $83.85\pm3.15^{b}$ | $13.46\pm3.38^{a}$ | $2.16\pm0.44^{b}$ | $0.53\pm0.09^{a}$ |
| **Combination** | $4.25\pm4.04^{a}$ | $87.59\pm7.44^{c}$ | $6.39\pm3.78^{b}$ | $1.77\pm0.91^{a}$ |

Superscript letters indicate statistical significance derived from two-way ANOVA and Tukey's multiple comparisons where different letters within the same column are statistically significant with P<0.0001, n=4.

**Table S4 Human apoptosis array coordinates and corresponding proteins**

| Coordinates | Protein | Coordinates | Protein |
|---|---|---|---|
| ***A1, A2*** | **Reference Spots1** | *C17, C18* | HSP60 |
| ***A23, A24*** | **Reference Spots2** | *C19, C20* | HSP70 |
| *B1, B2* | Bad | *C21, C22* | HTRA2/Omi |
| *B3, B4* | Bax | *C23, C24* | Livin |
| *B5, B6* | Bcl-2 | *D1, D2* | PON2 |
| *B7, B8* | Bcl-x | *D3, D4* | p21/CIP1/CDKN1A |
| *B9, B10* | Pro-Caspase-3 | *D5, D6* | p27/Kip1 |
| *B11, B12* | Cleaved Caspase-3 | *D7, D8* | Phospho-p53 (S15) |
| *B13, B14* | Catalase | *D9, D10* | Phospho-p53 (S46) |
| *B15, B16* | cIAP-1 | *D11, D12* | Phospho-p53 (S392) |
| *B17, B18* | cIAP-2 | *D13, D14* | Phospho-Rad17 (S635) |
| *B19, B20* | Claspin | *D15, D16* | SMAC/Diablo |
| *B21, B22* | Clusterin | *D17, D18* | Survivin |
| *B23, B24* | Cytochrome c | *D19, D20* | TNF RI/TNFRSF1A |
| *C1, C2* | TRAIL R1/DR4 | *D21, D22* | XIAP |
| *C3, C4* | TRAIL R2/DR5 | ***D23, D24*** | **PBS (Negative Control)** |
| *C5, C6* | FADD | | |
| *C7, C8* | Fas/TNFRSF6/CD95 | | |
| *C9, C10* | HIF-1$\alpha$ | | |
| *C11, C12* | HO-1/HMOX1/HSP32 | | |
| *C13, C14* | HO-2/HMOX2 | ***E1, E2*** | **Reference Spots3** |
| *C15, C16* | HSP27 | | |

**Table S5 Significant apoptotic proteins in MCF-7 cell lysate upon treatment by doxorubicin, propolis or its synergistic combination**

| Dysregulated proteins* & pairwise comparisons | p value | -Log10(p) | FDR | Fold Change | log2(FC) |
|---|---|---|---|---|---|
| *Doxorubicin vs control* | | | | | |
| Bcl-x | 0.000385 | 3.42 | 0.000923 | 0.32 | -1.63 |
| Claspin | 0.000345 | 3.46 | 0.000923 | 0.43 | -1.21 |
| Bad | 0.000117 | 3.93 | 0.000453 | 0.52 | -0.95 |
| Pro-Caspase-3 | 1.89E-07 | 6.72 | 3.31E-06 | 0.58 | -0.80 |
| Survivin | 0.000105 | 3.98 | 0.000453 | 0.59 | -0.75 |
| Cleaved Caspase-3 | 4.86E-06 | 5.31 | 5.68E-05 | 0.64 | -0.65 |
| cIAP-2 | 0.000593 | 3.23 | 0.001298 | 0.71# | -0.50 |
| Clusterin | 0.000961 | 3.02 | 0.001869 | 0.72# | -0.48 |
| Bcl-2 | 0.00023 | 3.64 | 0.000805 | 0.72# | -0.47 |
| Phospho-p53 (S15) | 0.002082 | 2.68 | 0.003644 | 1.36# | 0.45 |
| HO-1/HMOX1/HSP32 | 0.001036 | 2.98 | 0.001908 | 1.42 | 0.50 |
| Livin? | 0.000371 | 3.43 | 0.000923 | 1.43 | 0.52 |
| HO-2/HMOX2 | 0.000259 | 3.59 | 0.000824 | 1.51 | 0.59 |
| p21/CIP1/CDKN1A | 1.69E-05 | 4.77 | 0.000148 | 1.59 | 0.67 |
| Fas/TNFRSF6/CD95 | 0.000783 | 3.11 | 0.001612 | 1.64 | 0.71 |
| TRAIL R1/DR4 | 0.000115 | 3.94 | 0.000453 | 1.90 | 0.93 |
| TRAIL R2/DR5 | 7.04E-08 | 7.15 | 2.46E-06 | 2.37 | 1.25 |
| Phospho-p53 (S392) | 0.000396 | 3.40 | 0.000923 | 3.81 | 1.93 |
| *Propolis vs control* | | | | | |
| Bcl-x | 9.42E-05 | 4.03 | 0.000389 | 0.28 | -1.86 |
| Claspin | 0.000188 | 3.73 | 0.000689 | 0.41 | -1.29 |
| Pro-Caspase-3? | 1.54E-05 | 4.81 | 0.00017 | 0.50 | -1.00 |
| TNF RI/TNFRSF1A | 0.003504 | 2.46 | 0.007227 | 0.66 | -0.60 |
| Survivin | 9.33E-05 | 4.03 | 0.000389 | 0.67 | -0.58 |
| Phospho-p53 (S392)? | 0.012868 | 1.89 | 0.020221! | 0.71# | -0.50 |
| HIF-1 $\alpha$ | 0.001659 | 2.78 | 0.004211 | 1.32# | 0.41 |
| HSP70 | 2.10E-05 | 4.68 | 0.000173 | 1.35# | 0.43 |
| FADD | 4.36E-06 | 5.36 | 7.19E-05 | 1.40 | 0.48 |
| HTRA2/Omi | 0.000241 | 3.62 | 0.000796 | 1.66 | 0.73 |
| Catalase | 0.008252 | 2.08 | 0.013616! | 1.68 | 0.74 |
| TRAIL R1/DR4 | 0.000431 | 3.37 | 0.001292 | 1.89 | 0.92 |
| TRAIL R2/DR5 | 3.03E-05 | 4.52 | 0.0002 | 2.30 | 1.20 |
| HO-1/HMOX1/HSP32 | 1.64E-07 | 6.78 | 5.42E-06 | 2.84 | 1.51 |
| *Mixture vs (Doxorubicin and propolis)* | | | | | |
| XIAP | 0.001724 | 2.76 | 0.012071! | 0.71# | -0.49 |

| Dysregulated proteins* & pairwise comparisons | p value | -Log10(p) | FDR | Fold Change | log2(FC) |
|---|---|---|---|---|---|
| HSP60 | 0.006935 | 2.16 | 0.026969! | 0.71# | -0.49 |
| Cytochrome c | 0.00385 | 2.41 | 0.016845! | 0.72# | -0.47 |
| HIF-1$\alpha$ | 0.00381 | 2.42 | 0.016845! | 0.77# | -0.38 |
| cIAP-2 | 0.000396 | 3.40 | 0.00462 | 1.50 | 0.58 |
| p27/Kip1 | 0.001031 | 2.99 | 0.009018 | 1.50 | 0.58 |
| Claspin | 0.000272 | 3.57 | 0.00462 | 1.62 | 0.69 |
| PON2 | 3.11E-08 | 7.51 | 1.09E-06 | 1.88 | 0.91 |
| Catalase | 0.002956 | 2.53 | 0.016845! | 2.04 | 1.03 |

*red and blue coloured font of the proteins is significantly upregulated and downregulated respectively in its corresponding pairwise comparison. #; Slightly above the threshold for protein filtration,!; FDR>0.01.

**Table S6 Reactome overrepresented pathways using dysregulated proteins in MCF-7 cells treated with the synergistic propolis and doxorubicin combination**

| Pathway name | #Entities found | #Entities total | Entities pValue | Entities FDR | Rx found | Rx total | Rx ratio | Submitted entities found |
|---|---|---|---|---|---|---|---|---|
| TP53 Regulates Transcription of DNA Repair Genes | 3 | 89 | 1.88E-05 | 0.001636 | 3 | 17 | 0.001266 | BRCA1 |
| Transcriptional Regulation by E2F6 | 2 | 46 | 3.56E-04 | 0.015304 | 2 | 33 | 0.002458 | BRCA1 |
| Ion transport by P-type ATPases | 2 | 71 | 8.41E-04 | 0.017343 | 2 | 15 | 0.001117 | ATP8B4 |
| Diseases of DNA Double-Strand Break Repair | 1 | 2 | 0.001239 | 0.017343 | 2 | 2 | 1.49E-04 | BRCA1 |
| Defective DNA double strand break response due to BRCA1 loss of function | 1 | 2 | 0.001239 | 0.017343 | 1 | 1 | 7.45E-05 | BRCA1 |
| Transcriptional Regulation by TP53 | 3 | 487 | 0.002721 | 0.03265 | 4 | 259 | 0.019291 | BRCA1 |
| TP53 Regulates Transcription of DNA Repair Genes | 3 | 89 | 2.07E-04 | 0.02227 | 3 | 17 | 0.001266 | MDC1 |
| Transcriptional Regulation by TP53 | 5 | 487 | 3.32E-04 | 0.02227 | 5 | 259 | 0.019291 | MDC1;CHD3;COX6B1 |

*Upregulated and downregulated proteins are red and blue coloured, respectively.

**Table S7 IMPala overrepresented pathways using dysregulated proteins in MCF-7 cells treated with the synergistic propolis and doxorubicin combination**

| pathway_name | pathway_source | genes | overlapping_genes | num_all_pathway_genes | P_genes | Q_genes |
|---|---|---|---|---|---|---|
| brca1 dependent ub ligase activity | BioCarta | 1 | BRCA1 | 8 (8) | 0.00308 | 1 |
| Transcriptional Regulation by E2F6 | Wikipathways | 1 | BRCA1 | 10 (10) | 0.00384 | 1 |
| TP53 Regulates Transcription of DNA Repair Genes | Wikipathways | 1 | BRCA1 | 14 (14) | 0.00538 | 1 |
| PI3K-Akt Signaling Pathway | Wikipathways | 2 | BRCA1;LAMA4 | 339 (340) | 0.00644 | 1 |
| atm signaling pathway | BioCarta | 1 | BRCA1 | 18 (18) | 0.00691 | 1 |
| MET activates PTK2 signaling | Reactome | 1 | LAMA4 | 18 (18) | 0.00691 | 1 |
| PI3K-Akt signaling pathway - Homo sapiens (human) | KEGG | 2 | BRCA1;LAMA4 | 353 (354) | 0.00697 | 1 |
| prion pathway | BioCarta | 1 | LAMA4 | 19 (19) | 0.00729 | 1 |
| cell cycle: g2/m checkpoint | BioCarta | 1 | BRCA1 | 21 (21) | 0.00806 | 1 |
| role of brca1 brca2 and atr in cancer susceptibility | BioCarta | 1 | BRCA1 | 22 (22) | 0.00844 | 1 |
| Laminin interactions | Reactome | 1 | LAMA4 | 23 (23) | 0.00882 | 1 |
| Antigen Presentation: Folding_ assembly and peptide loading of class I MHC | Reactome | 1 | SEC23A | 24 (24) | 0.0092 | 1 |
| ATM Signaling Network in Development and Disease | Wikipathways | 2 | MDC1;RIF1 | 46 (46) | 0.000661 | 1 |
| Nonhomologous End-Joining (NHEJ) | Reactome | 2 | MDC1;RIF1 | 69 (70) | 0.00148 | 1 |
| Transcriptional Regulation by TP53 | Reactome | 3 | MDC1;CHD3;COX6B1 | 371 (374) | 0.00321 | 1 |
| SUMO E3 ligases SUMOylate target proteins | Reactome | 2 | MDC1;CHD3 | 120 (120) | 0.00441 | 1 |
| SUMOylation | Reactome | 2 | MDC1;CHD3 | 126 (126) | 0.00485 | 1 |
| DNA Double-Strand Break Repair | Reactome | 2 | MDC1;RIF1 | 167 (169) | 0.00837 | 1 |

*Upregulated and downregulated proteins are red and blue coloured, respectively.

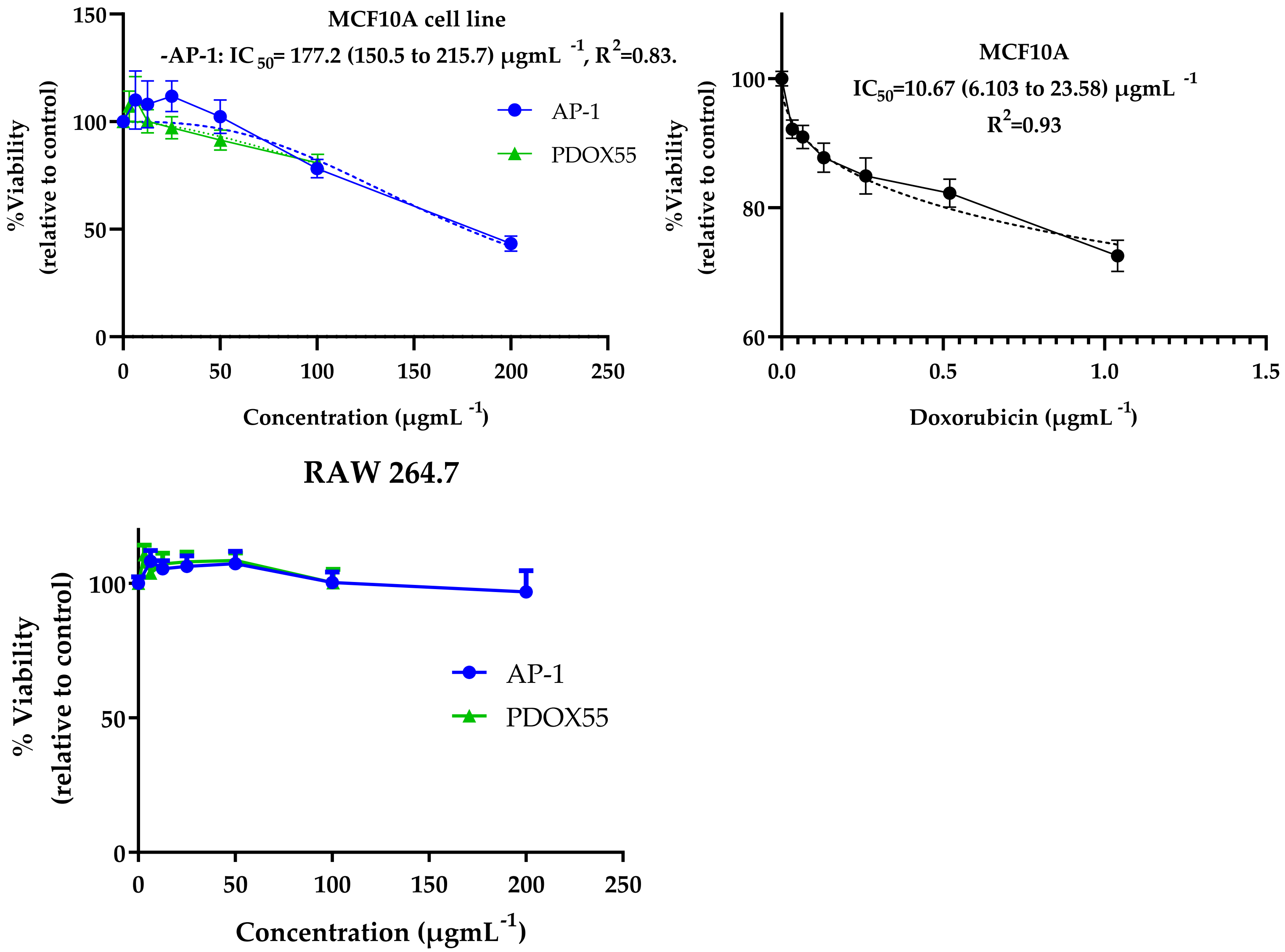


**Figure S1 Viability of RAW 264.7 and MCF10A cells upon treatment with AP-1 and PDOX55 along the dose-response curve of DOX against MCF10A cells.**

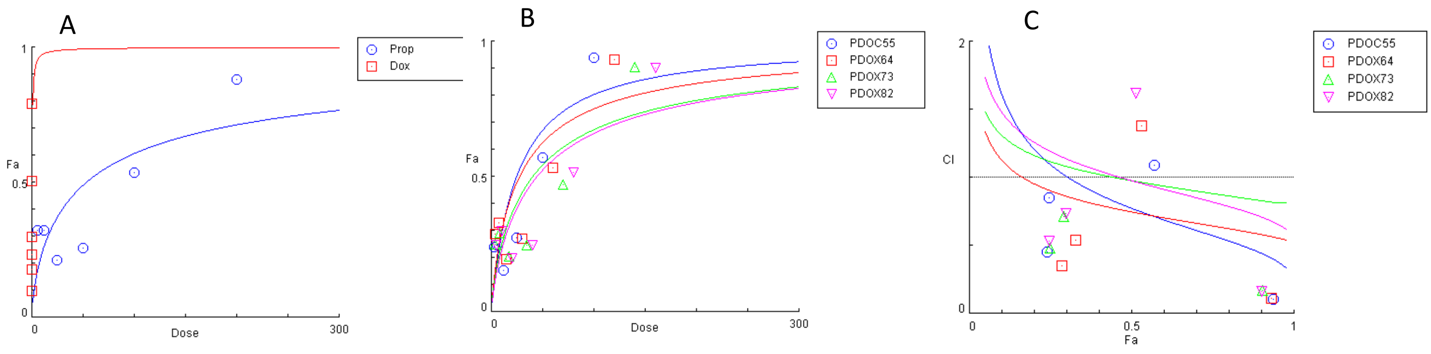


**Figure S2 Dose-response curves of (A) Australian propolis (Prop), doxorubicin (Dox) and most synergistic combinations (B) together with its combination index (CI) plot (C) calculated from CompuSyn software.**

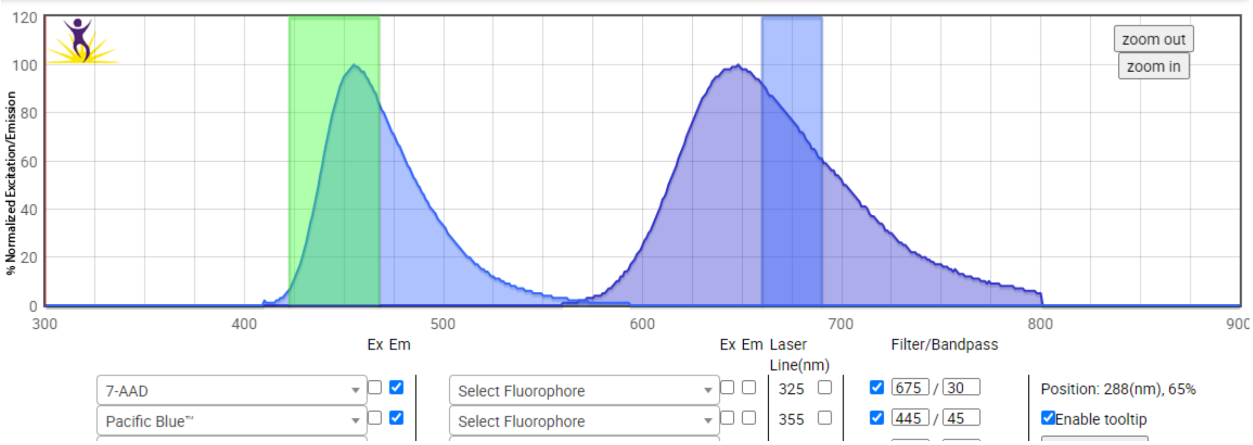


**Figure S3 Emission spectra for Annexin V and 7-AAD using PerCP and Pacific blue channels ([https://www.biolegend.com/en-us/spectra-analyzer](https://www.biolegend.com/en-us/spectra-analyzer))**

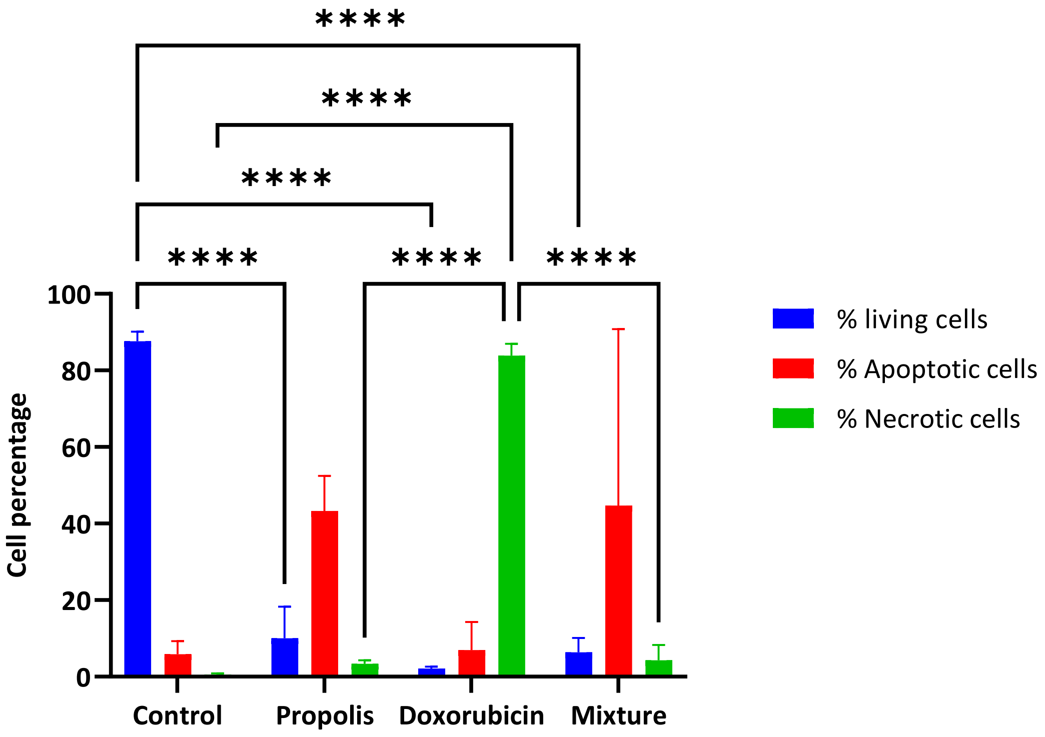


**Figure S4 Combined cell percentage analysis in different treatment groups in quadruplicates. Twenty-four hours treatment of propolis extract (100 μgmL$^{-1}$), Dox; doxorubicin (0.22 μgmL$^{-1}$), and their mixture at a half-dose synergistic combination (50 μgmL$^{-1}$ and 0.11 μgmL$^{-1}$, respectively) with a negative control were implemented using antibodies against Annexin-V CF-Blue and the reporter 7AAD. ****; significantly different as derived from Two-way ANOVA and Tukey's multiple comparisons at $P<0.001$, n=4.**

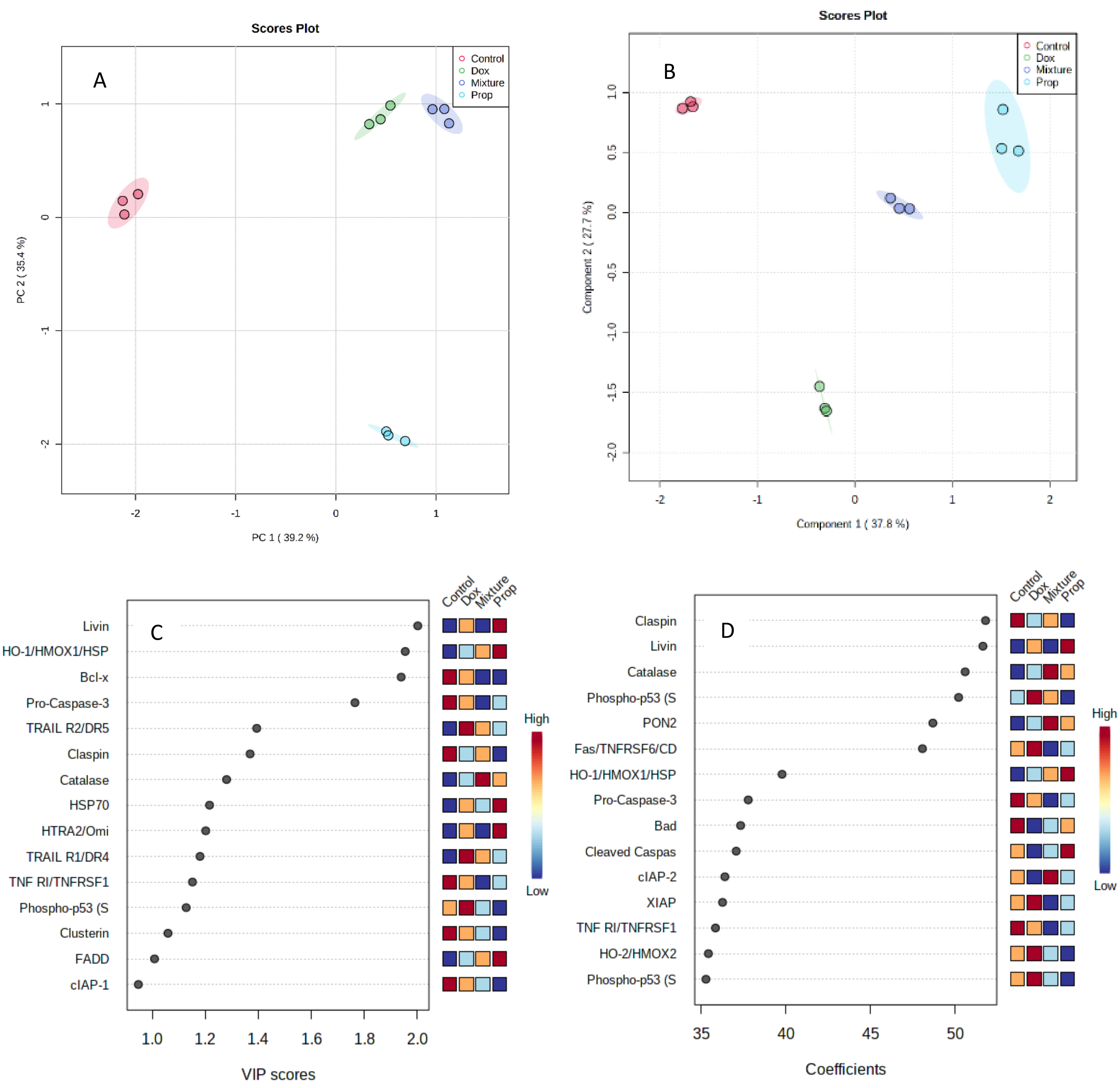


**Figure S5 Supervised PLS-DA and Unsupervised PCA multivariate data analysis of the apoptotic proteins of MCF-7 lysates analysed by Proteome profiler human apoptotic array kit. (A) Score plot of principal component analysis (PCA) between the selected principal components alongside the explained variances shown in brackets. (B) Score plot of partial least square discriminant analysis (PLS-DA) where third averaged point was imputed for each treatment, (C and D) are the most discriminatory proteins in among different treatments identified by variable importance projection (VIP) score and coefficients of PLS-Da model, respectively. The coloured boxes on the right indicate the relative concentrations of the corresponding metabolite in each group under study.**

**A**

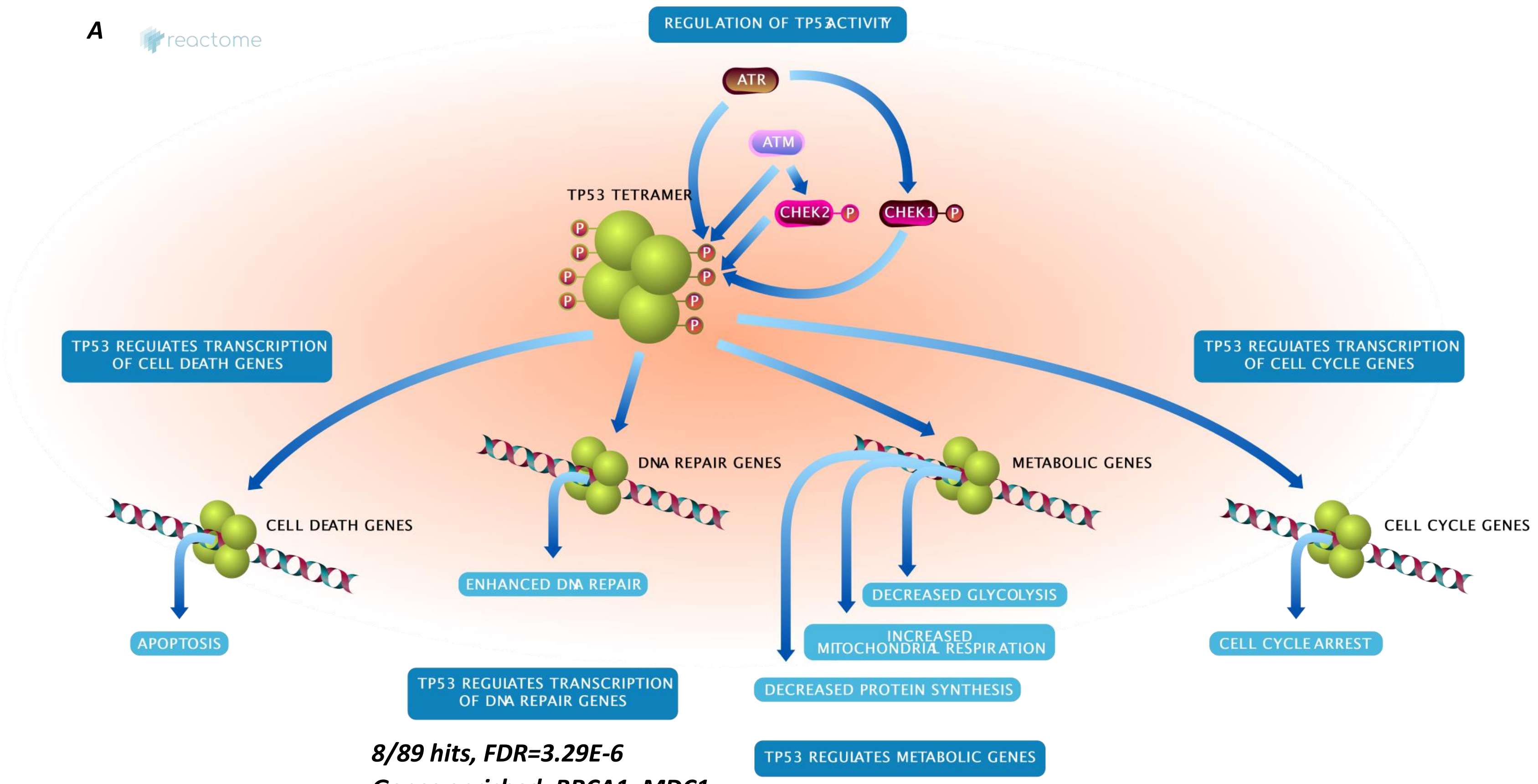


***8/89 hits, FDR=3.29E-6***

***Genes enriched; BRCA1, MDC1***

**Figure S6 Reactome downloaded TP53 transcription regulation pathway (A), TP53 transcriptional regulation of DNA repair genes with the enriched genes of BRCA1 and MCD1 marked by an orange star.**

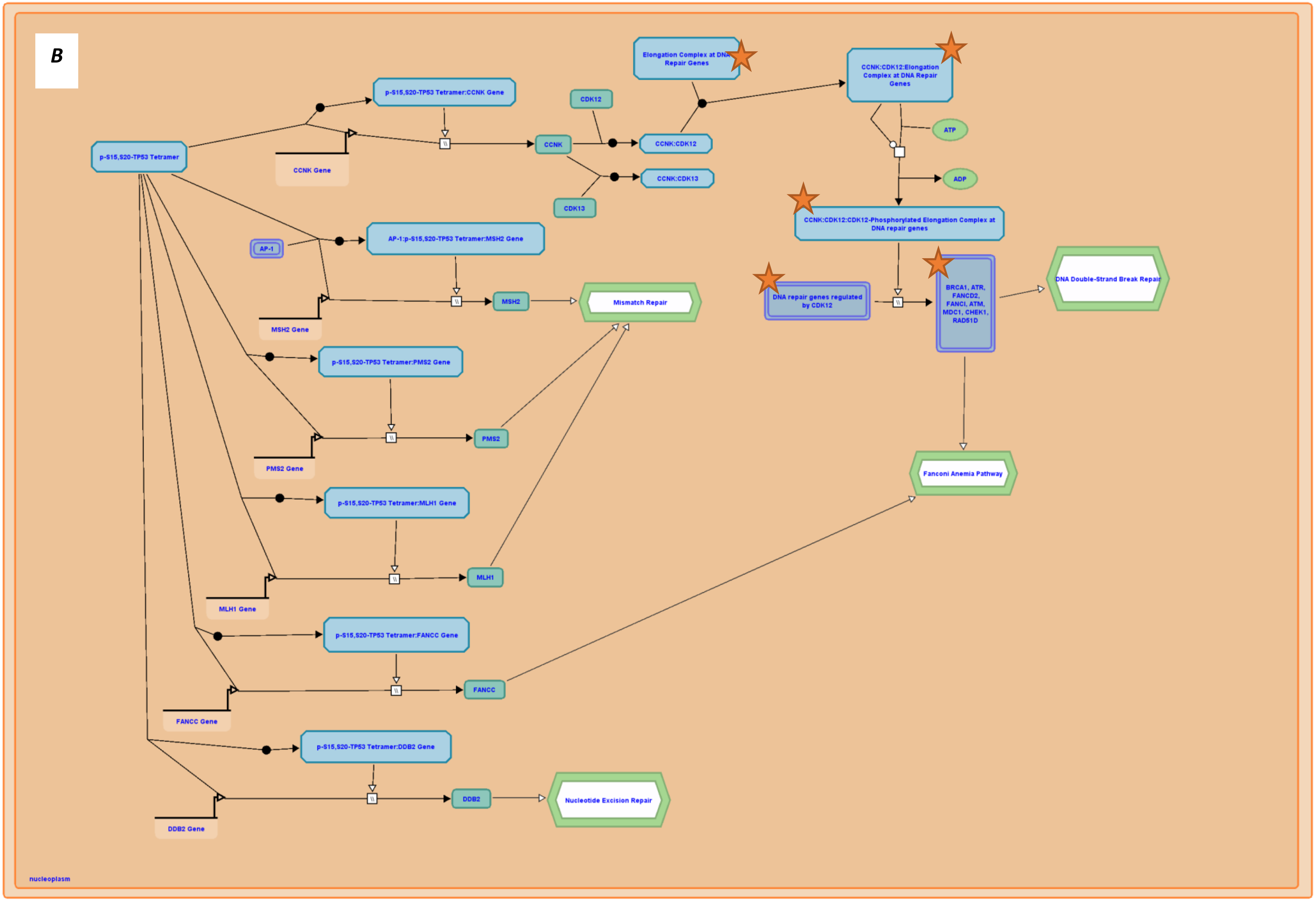


*Figure S6 continued*

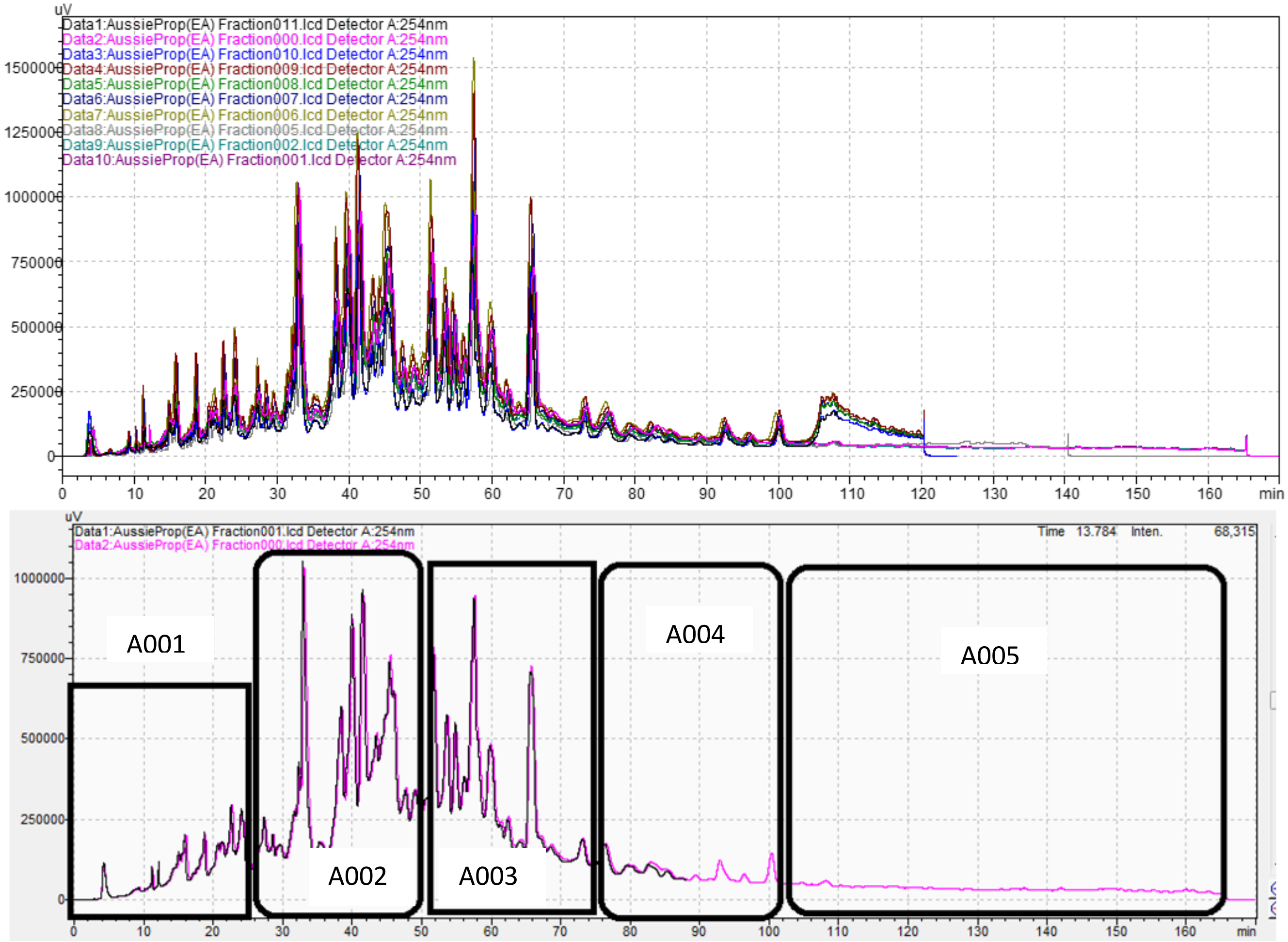


**Figure S7 Preparative HPLC fractionation of Australian propolis extract**
